# A single short reprogramming early in life initiates and propagates an epigenetically related mechanism improving fitness and promoting an increased healthy lifespan

**DOI:** 10.1111/acel.13714

**Published:** 2022-10-17

**Authors:** Quentin Alle, Enora Le Borgne, Paul Bensadoun, Camille Lemey, Nelly Béchir, Mélissa Gabanou, Fanny Estermann, Christelle Bertrand‐Gaday, Laurence Pessemesse, Karine Toupet, Romain Desprat, Jérôme Vialaret, Christophe Hirtz, Danièle Noël, Christian Jorgensen, François Casas, Ollivier Milhavet, Jean‐Marc Lemaitre

**Affiliations:** ^1^ IRMB, Univ Montpellier, INSERM Montpellier France; ^2^ DMEM, Univ Montpellier, INRAE Montpellier France; ^3^ RAM‐METAMUS, Univ Montpellier, INRAE Montpellier France; ^4^ ECELLFrance Montpellier Facility, Univ Montpellier Montpellier France; ^5^ SAFE‐iPSC Facility, CHU Montpellier Montpellier France; ^6^ PPC Facility, CHU Montpellier Montpellier France; ^7^ IRMB, Univ Montpellier, INSERM, CNRS Montpellier France

**Keywords:** aging, epigenetics, longevity, metabolism, transient reprogramming

## Abstract

Recent advances in cell reprogramming showed that OSKM induction is able to improve cell physiology in vitro and in vivo. Here, we show that a single short reprogramming induction is sufficient to prevent musculoskeletal functions deterioration of mice, when applied in early life. In addition, in old age, treated mice have improved tissue structures in kidney, spleen, skin, and lung, with an increased lifespan of 15% associated with organ‐specific differential age‐related DNA methylation signatures rejuvenated by the treatment. Altogether, our results indicate that a single short reprogramming early in life might initiate and propagate an epigenetically related mechanism to promote a healthy lifespan.

## Graphical abstract

Our results indicated that a single short reprogramming early in life promote a healthy lifespan in mice. Indeed, a single induction with Yamanaka's factors (OSKM) was sufficient to prevent musculoskeletal functions deterioration, when applied in early life. In old age, treated mice have improved tissue structures with an increased lifespan. These improvements were associated with organ‐specific differential age‐related DNA methylation signatures after OSKM induction and propagated throughout life conceivably fueling epigenetic rejuvenation observed in aged animals.

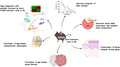

## 1 INTRODUCTION

Over the course of a human lifetime, the entry into old age brings increased likelihood of contracting age‐related diseases. The aging demographic makes this issue a central scientific concern in medicine. Aging is a complex process often punctuated by the appearance of age‐related pathologies and a decrease of cell and tissue regenerative capacity. It intensifies cell and tissue vulnerability and deterioration and increases the risk of developing diseases like cancer, cardiovascular disorders, diabetes, atherosclerosis, age‐related macular degeneration or neurodegeneration and ultimately precipitating death (Campisi, 2013; López‐Otín et al., 2013). The mechanisms causing aging are still poorly understood, making it difficult to develop prophylactic strategies to increase healthy lifespan. There are numerous molecular and cellular hallmarks of the aging process, including cellular senescence, genomic instability, deregulated autophagy, mitochondrial dysfunction, telomere shortening, oxidative stress, systemic inflammation, metabolism dysfunctions, epigenetic alterations, and stem cell exhaustion (López‐Otín et al., 2013). Although many of these hallmarks have been extensively described and studied, few of them have been translated into effective therapies, with the notable exception of the removal of senescent cells, which has led to the development of senolytic drugs in humans (Childs et al., 2017). Among the described hallmarks, DNA methylation was proposed to be pertinent to evaluate the physiological age of individuals, since the deviation of predicted and chronological age correlates with all‐cause mortality in human (Horvath & Raj, 2018; Marioni et al., 2015) and was described to be affected by genetic, dietary, or pharmacological interventions (Field et al., 2018).

In 2006 and 2007, it was shown that adult cells from mice and human can be converted into pluripotent cells (iPSCs) by the overexpression of four transcription factors OCT4, SOX2, KLF4, and CMYC (Takahashi et al., 2007; Takahashi & Yamanaka, 2006). This process of cellular reprogramming induces a global remodeling of epigenetic landscape to revert cell identity into a pluripotent embryonic‐like state. Although cellular senescence and aging were described as a limitation to iPSC derivation (Banito et al., 2009; Hong et al., 2009; Kawamura et al., 2009; Li et al., 2009; Marión et al., 2009; Utikal et al., 2009), we developed an optimized iPSC‐derived strategy to rejuvenate senescent cells and cells from centenarians individuals through the pluripotent state, using two additional factors NANOG and LIN28 (OSKMNL), demonstrating that cellular aging was reversible (Lapasset et al., 2011). Recently, a new reprogramming method was developed using a transient expression of a this OSKMNL factors promoting amelioration of aging hallmarks in human cells in vitro (Sarkar et al., 2020). Although, initially, in vivo OSKM expression in mice models was described to generate teratomas leading to death in mice (Abad et al., 2013), a transient expression protocol has been recently developed, demonstrating that a cyclic induction of OSKM 2 days a week (thus avoiding continuous expression), over the entire extremely short lifetime of a homozygous accelerated aging mouse model, increased longevity (Ocampo et al., 2016). OSKM induction also ameliorated immediate tissue regeneration after experimentally induced injury and various local reprogramming protocols were applied to different mice models to study the immediate impact on tissue fitness. Consequently, direct or indirect beneficial or deleterious effect in different tissues was reported (Browder et al., 2022; Chen et al., 2021; Chiche et al., 2017; Chondronasiou et al., 2022; Doeser et al., 2018; Lu et al., 2020; Mosteiro et al., 2016; Ocampo et al., 2016; Ohnishi et al., 2014; Olova et al., 2019; Rodríguez‐Matellán et al., 2020; Senís et al., 2018; Wang et al., 2021). However, how OSKM induction might increase lifespan in preventing tissues aging and age‐related diseases is still an unresolved issue.

In this study, we explore the window of efficacy of OSKM reprogramming by concentrating on heterozygous animals, which have moderate lifespan and levels of progerin (Osorio et al., 2011), as these heterozygotes might be extremely sensitive to anti‐aging therapies. In this study, we investigate for the impact of a single short period of in vivo OSKM induction on aging, as pre‐clinical proof of principle for a potential usage in clinic to prevent aging defects. We found that cyclic induction of OSKM improved life span of the heterozygotes as expected, but that a continuous lower induction worked equally well. To further test the requirements, we wondered if a short window of treatment could have similar effects to cyclic or continuous lifelong treatment. Surprisingly, we found that age‐related tissue deterioration and longevity itself were ameliorated in elderly, by a single two and a half weeks treatment on two‐month‐old mice. We identified organ‐specific differential DNA methylation signatures related to aging partially prevented by our specific protocol modifying positively this epigenetic drift in old age. Consequently, our results indicate that a single short reprogramming early in life might initiate and propagate an epigenetically related mechanism, to promote a healthy lifespan.

## 2 RESULTS AND DISCUSSION

### 2.1 A single early short transient reprogramming increases late age lifespan

Previous experiments demonstrated that a cyclic induction of OSKM 2 days a week, over the entire extremely short lifetime of the homozygous accelerated aging mouse model (Lmna^G609G/G609G^), increased longevity (Ocampo et al., 2016). These mice have two alleles of a mutated *Lmna* gene, producing high level of the protein progerin, a truncated form of lamin A, recapitulating the human phenotype of HGPS, leading to short‐lived mice (Osorio et al., 2011).

As progerin is produced at low level in natural aging (Scaffidi & Misteli, 2006), we considered a specific mouse model (Lmna^G609G/+^) producing less progerin, to be closer to physiological aging. Whereas homozygous mice have an average lifespan of 15 weeks, heterozygous mice live around 35 weeks, presenting accelerated onset of several phenotypic alterations related to aging (Osorio et al., 2011). We crossed these mice with a homozygous transgenic murine model (R26^rtTA/rtTA^;Col1a1^4F2A/4F2A^) allowing the controlled induction of the expression of OSKM factors through an rtTA transactivator, by addition of doxycycline (DOX) in the drinking water, thus reprogramming all the animal's cells (Carey et al., 2010).

**FIGURE 1:**
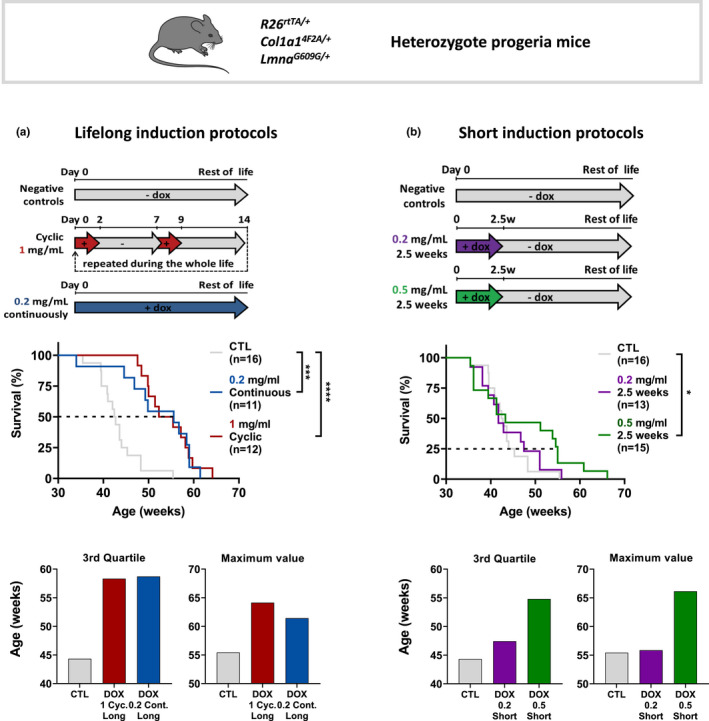
Single short transient OSKM induction applied early in life, increases late age lifespan in heterozygous progeric mice. (a) Scheme of the long‐term OSKM induction protocols for R26^rtTA/+^; Col1a1^4F2A/+^; Lmna^G609G/+^ heterozygous progeric mice, by administrating 1 mg/ml doxycycline in the drinking water chronically 2 days a week (red curve), started at 2 months old and maintained over the entire life, or 0.2 mg/ml docycycline administrated continuously (blue curve). Survival curves of long‐term doxycycline‐treated mice compared to untreated mice (gray curve) with the same genotype are presented. Statistical analysis of curves was performed at the corresponding indicated percent survival. Median survival for the 3rd quartile and maximum lifespan value is presented. (b) Short‐term OSKM induction protocols were performed on progeric R26^rtTA/+^;Col1a1^4F2A/+^;Lmna^G609G/+^ mice, by administrating doxycycline in the drinking water for 2.5 weeks either at 0.2 mg/ml (purple curve) or at 0.5 mg/ml (green curve). Inductions start at 2 months old. Survival curves of short‐term doxycycline‐treated mice after induction compared to untreated mice with the same genotype are presented. Statistical analysis of curves was performed at the corresponding indicated percent survival. Median survival for the 3rd quartile and maximum lifespan value is presented. *p = 0.0113, ***p = 0.0003, ****p < 0.0001; according to log‐rank (mantel‐cox) test.

Here, we sought to characterize the heterozygous progeric mice, as a sensitive and physiological aging model and also to revisit, explore and simplify the previously published OSKM treatment, which is to administrate 1 mg/ml DOX, 2 days a week, throughout life (Ocampo et al., 2016). Thus, using this protocol as a positive control, we tested a simplified protocol, where a reduced concentration of 0.2 mg/ml was added continuously throughout life. Strikingly, this simplified protocol gave near identical improvement to heterozygous lifespan, with a high increase in median age of death, from 42.6 to 55.6 weeks, meaning that lower doses of doxycycline could be effective (Figure 1a).

In parallel, we further speculated that a single period of treatment might cause a permanent improvement in vitality and we designed an extremely simplified protocol since it might be straightforward for clinical translation. We thus induced reprogramming factors with 0.2 mg/ml and 0.5 mg/ml DOX for just a single two and a half weeks treatment at 2 months of age. There was a very minor but non‐significant effect at 0.2 mg/ml, whereas the more concentrated treatment at 0.5 mg/ml DOX noticeably improved lifespan in those mice (Figure 1b). Indeed, while no difference was noticeable in median age of death, the 0.5 mg/ml doxycycline treatment remarkably increased the age of death for the third quartile from 44.3 weeks to 54.8 weeks for treated animals compared to controls. Surprisingly, this protocol also increased the maximum lifespan in the group to 66.1 weeks, which is 11 weeks longer than the longest‐lived animal (Figure 1b). Similarly, induction protocol at 0.5 mg/ml DOX for just a single two and a half weeks treatment at 2 months of age in non‐progeric mice led to a similar increased lifespan in old age (Figure S1).

These results indicate for the first time that a single short transient expression of reprogramming factors in vivo may increase lifespan both in accelerated and natural aging mice.

### 2.2 A single short reprogramming treatment ameliorates body composition and motor skills

**FIGURE 2:**
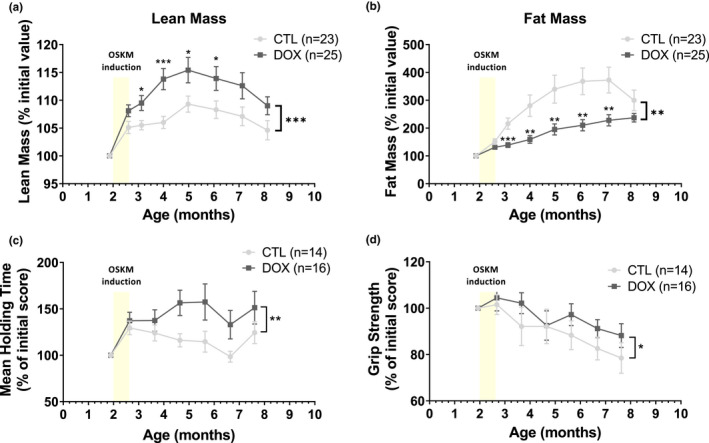
Single OSKM induction early in life induces healthier body composition and improves lifelong muscular capacities in progeric mice. (a) Body lean mass composition of treated heterozygotes progeric mice with 0.5 mg/ml doxycycline (DOX) for 2.5 weeks at the age of 2 months, compared with untreated controls (CTL) measured by EchoMRI‐700. Results are expressed in percentage of total individual weight. *p‐value <0.05, ***p‐value <0.001 according to multiple *t* test for 1 vs 1 comparisons and paired *t* test for whole curves. (b) Body fat mass composition through over life measured by EchoMRI‐700. Results are expressed in percentage of total individual weight. **p‐value <0.01, ***p‐value <0.001 according to multiple *t* test for 1 vs 1 comparisons and paired *t* test for whole curves. (c) Rotarod assay. Maximal time to fall (strength endurance) compared to initial individual score. **p‐value <0.01 is according to paired *t* test on whole curves. (d) Maximal grip strength compared to initial individual score. *p‐value <0.05 is according to paired *t* test on whole curves. The period of treatment in mentioned in each graph as a yellow bar ±DOX.

To gain further insight of the impact of an early treatment on aging, we decided to study the potential effect of the single short reprogramming induction protocol on organismal metabolism and its consequences. Maintenance of lean mass and mobility is a pertinent indicator of health, both in human and mice (Kyle et al., 2001). Unexpectedly, while no significant effect on the total body weight evolution was revealed (data not shown), we observed a highly significant higher lean mass proportion in treated progeric animals, starting early after the treatment and maintained during aging (Figure 2a). Consistently, a highly significant lower percentage of fat mass was observed suggesting that a global metabolic switch is triggered by our short reprogramming protocol, early in life, expressing the OSKM cassette in white adipose tissue and muscle. This leads to the amelioration of body composition in treated animal (Figure 2b), whereas reprogramming factors are downregulated (Figure S2a).

As it generally favors healthy aging, we also evaluated the motor coordination of treated animals using the traditional increasing speed method on a rotarod (Justice et al., 2014) and we also tested muscle strength by grip tests. Both tests revealed improved motor skills in treated animal, initiated early after the treatment and maintained in aging (Figure 2c,d). Recently, a local chronic OSKM induction regimen in muscle was described to improve regeneration after injury by remodeling the muscle stem cell niche (Wang et al., 2021), refining previous observations suggesting a role of Pax7 muscle stem cell (Ocampo et al., 2016). Although this piece of work identified an immediate improvement of muscle regeneration after injury, it did not show long‐term effect, as recently confirmed in a very recent study (Browder et al., 2022).

Consequently, our results demonstrate that a single short reprogramming induction early in life can initiate lifelong improvement of body composition, with positive consequences on motor skills maintained during aging.

### 2.3 A single short reprogramming treatment prevents age‐related tissue structure deteriorations and fibrosis

With a view to deepen our understanding of the effects of the transient reprogramming enabled by our short treatment, we extended our analysis to a wide panel of organs to highlight macroscopic changes in their structure and integrity.

**FIGURE 3:**
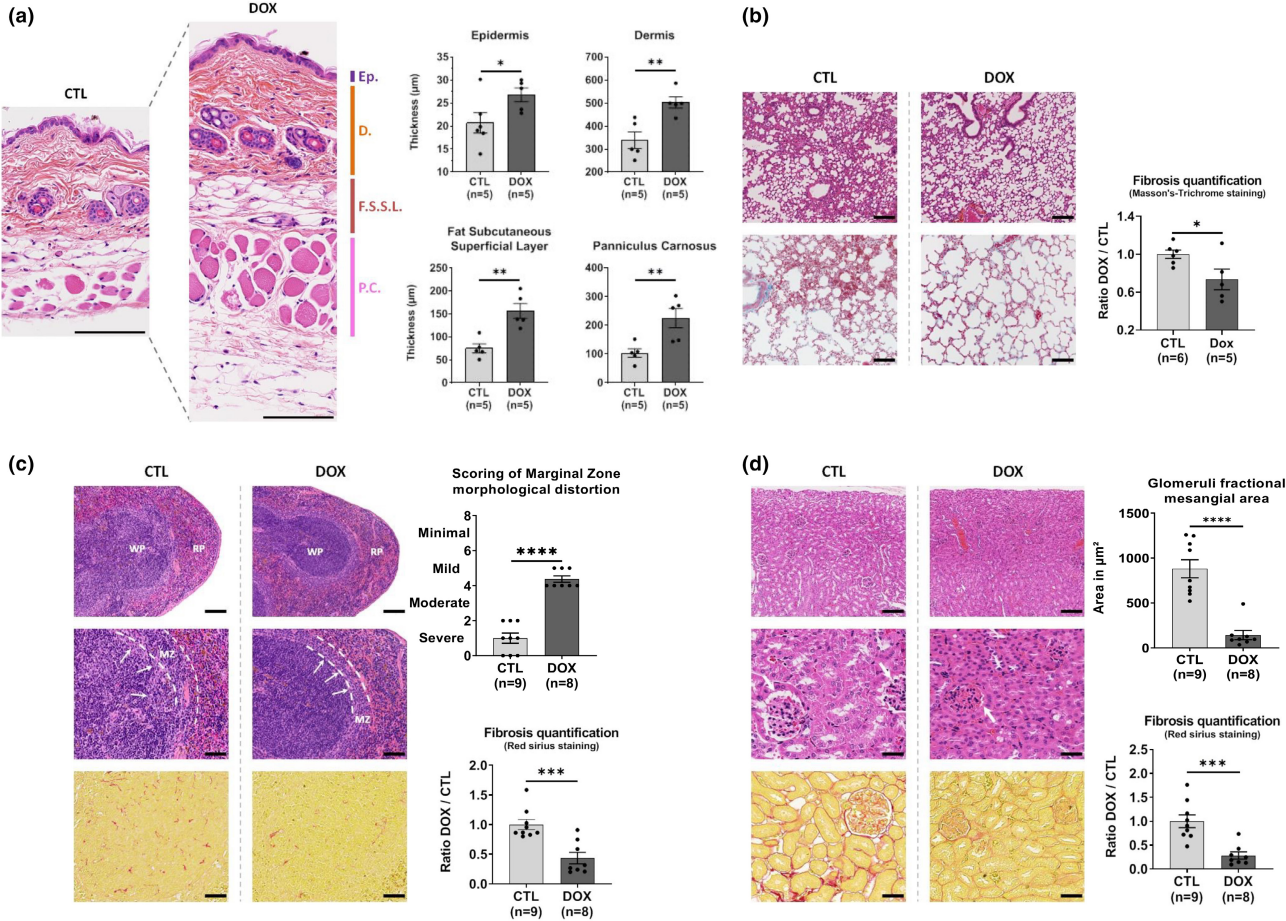
Tissue structure and age‐related tissue fibrosis are improved in aging by a single OSKM induction early in life. (a) Quantifications of skin layers thickness. D., dermis; Ep., epidermis; F.S.S.L., fat subcutaneous superficial layer; P.C., panniculus carnosus. HES staining, 150 μm scale. (b) Morphologic comparison of lung structure (on top) and fibrosis (on bottom) in treated and untreated mice. Scoring of fibrosis level is as described in methods. On top: HES staining, 150 μm scale. On bottom: MT staining, 75 μm scale. (c) Morphologic comparison of spleen marginal zone (MZ) architecture (on top and middle) and white pulp fibrosis (on bottom). The MZ/white pulp interface distortion is depicted by the inner line and the percent of radius involvement (MZ protruding into the white pulp area) is depicted by arrows and scoring was determined as described methods. On top: HES staining, 150 μm scale. In middle: HES staining, 75 μm. On bottom: SR staining, 37.5 μm scale. (d) Measurement of kidney's fractional mesangial area (on top and middle), representing the space surrounding glomeruli (depicted by arrows) and inter‐tubular fibrosis (on bottom). On top: HES staining, 75 μm scale. In middle: HES staining, 37.5 μm. On bottom: SR staining, 37.5 μm scale. All tissues were analyzed on 8‐month‐old heterozygous progeric mice. CTL represents untreated mice and DOX represents treated mice with 0.5 mg/ml doxycycline for 2.5 weeks at the age of 2 months. All Measurements of areas and distances were performed on ImageJ software. The fibrosis was scored as described in the methods. ****p < 0.0001; ***p < 0.001; **p < 0.01; *p < 0.05 is according to unpaired *t* test, two‐tailed.

Skin gradually loses its self‐renewal potential during aging and becomes thinner, losing its properties of resistance, plasticity, and elasticity, as well as its role of protective barrier (Zouboulis & Makrantonaki, 2011). Strikingly, our short reprogramming protocol at 0.5 mg/ml DOX induced at 2 months of age led to a major protective effect on skin age‐related thickness atrophy with a positive impact on all skin layers observed at 8 months of age (Figure 3a). There was a 40% average thickening of the epidermis and dermis, while the fat subcutaneous superficial layer and the panniculus carnosus smooth muscle layer increased by 120%. Thus, this result demonstrates that the short reprogramming in the early life is able to delay skin deteriorations resulting in a maintained integrity at up to 8 months of age.

Idiopathic pulmonary fibrosis, most often observed in patient over 70 years of age, is a severe pulmonary impairment resulting in death, 2 to 5 years after diagnosis, from respiratory failure. Fibrosis results in excessive accumulation of extracellular matrix and remodeling of lung architecture, in particular with the filling of the alveolar space with connective tissue (Noble et al., 2012). To explore the impact of our short reprogramming on lung alterations and fibrosis, we used the validated Masson's Trichrome staining. Quantification of fibrosis, in mice treated by our short early‐in‐life reprogramming protocol, showed significant decrease of covered areas at 8 months of age, suggesting a protective effect on age‐related pulmonary deterioration (Figure 3b).

Spleen, as well as lymph nodes, is an important secondary lymphoid organ involved in immune response to pathogens and prevention of senescent cells accumulation during aging (Palacio et al., 2019). Spleen structural loss of integrity is commonly observed in the elderly, compromising the immune system efficiency. Histological analysis of the spleen revealed significant changes induced by our treatment early in life. Indeed, after scoring the architecture of the marginal zone, as presented in methods, we determined that the treated animals obtained an average score ranging from 4 to 6, characterizing a mild alteration against an average score ranging from 0 to 2 characterizing a highly significant severe alteration of the marginal zone in the control animals (Figure 3c). In addition, we measured a decreased fibrosis in treated mice, confirming that our short OSKM induction protocol, early in life, led to a greater maintenance of spleen integrity in elderly.

Kidneys are also affected during aging where a loss of integrity and increased fibrosis alters their functions, and may result in an increased susceptibility to drug toxicity and a potentially harmful electrolyte imbalance. The histological features from the elderly include decreased cortical mass, glomerulosclerosis, interstitial fibrosis, tubular atrophy, and arteriosclerosis. Our study of the renal tissue first focused on the space surrounding the glomeruli, also named the fractional mesangial area and which is known to increase during aging (Lim et al., 2012). In the group of treated mice, we measured a strong significant decrease of the fractional mesangial area of 85% on average with an associated decreased fibrosis (Figure 3D).

Thus, in addition to the amelioration of organ structure, a global decrease of fibrosis at the organismal level is observed and particularly significant in lungs, spleen, and kidneys but also with a downward trend in liver and heart (Figure S3).

Consequently, our data clearly demonstrated that structure and integrity of several tissues, in older individuals, are positively impacted by a single short reprogramming, through the early expression of the OSKM cassette (Figure S2b).

### 2.4 A single short reprogramming treatment prevents age‐related osteoarticular diseases

**FIGURE 4:**
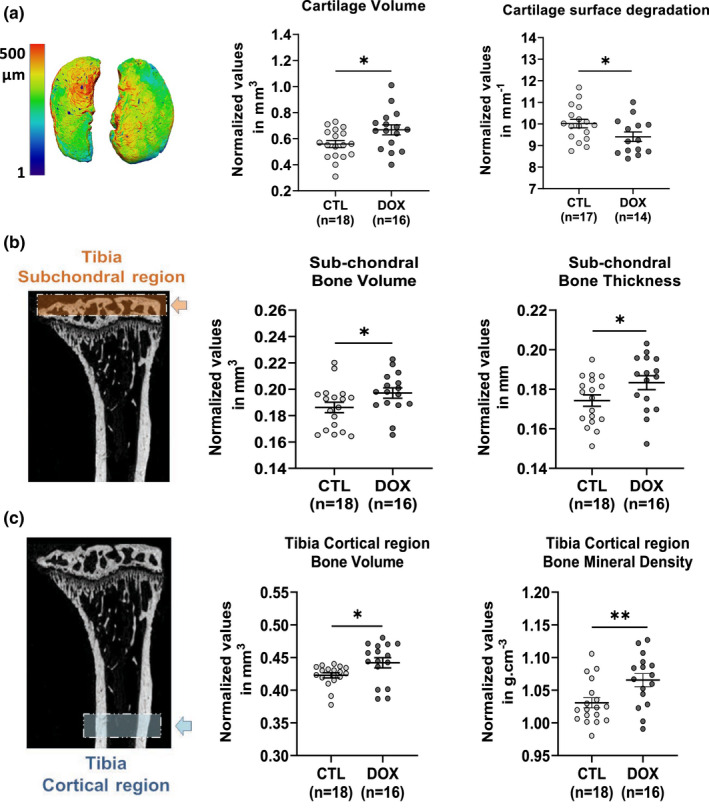
Single short OSKM induction early in life prevents osteoarthritis and osteoporosis in aged mice. (a) Histomorphometric analysis of 3D images of knee joint cartilage by confocal laser scanning microscopy (CLSM). Cartilage volume, and surface degradation were measured in the lateral and medial plateau. *p < 0.05 is according to unpaired *t* test, two‐tailed. (b) X‐ray micro‐computed tomography (μ‐CT). Histomorphometric analysis of left tibia subchondral bone, in the knee joint (orange box and arrow). Subchondral bone volume and thickness were measured in the lateral and medial plateau. (c) μ‐CT histomorphometric analysis of tibia cortical region (blue box and arrow). Cortical bone volume and mineral density were measured on both tibias. Bone and cartilage tissues were analyzed on 8 months heterozygous progeric mice. CTL represents untreated mice and DOX represents treated mice with 0.5 mg/ml doxycycline during 2.5 weeks at the age of 2 months. For μCT analysis, **p < 0.01; *p < 0.05 is according to unpaired *t* test, one‐tailed, with Welch's correction.

Osteoarthritis is the most common joint disease in humans, and etiology of the pathology is a complex combination of hereditary and environmental determinants (Hunter & Bierma‐Zeinstra, 2019). At the knee level, it is characterized by an alteration of all the structures of the joint and in particular by a degradation of the cartilage which can even go as far as a degradation of the subchondral bone. We thus wanted to assess whether there was an improvement in cartilage and bone tissue in the knees of our treated mice (Figure 4). We studied the cartilage of the lateral and median plates of the left knee by confocal laser scanning microscopy (CLSM), and the subchondral bone of these same plates was analyzed by X‐ray microtomography (μ‐CT). The CSLM measurements revealed that the mice induced at the age of 2 months had a significantly higher volume of cartilage and a reduced cartilage surface degradation 6 months later, when compared to untreated control mice (Figure 4a). This trend was confirmed in the subchondral bone by a decrease in the degradation of the surface exemplified by a smoother appearance of the bone on the 3D reconstruction (Figure S4a).

Another pathology commonly encountered during aging is osteoporosis. This skeletal pathology decreases bone mass and deteriorates its internal structure making it more fragile, and greatly increases the risk of fracture (Aspray & Hill, 2019). The causes of osteoporosis are manifold and still mostly unknown. However, consequences of subchondral bone structure alterations associated with osteoporosis might be an early event in osteoarthritis pathology (Stewart & Kawcak, 2018). To assess the level of osteoporosis in our animals, we firstly analyzed the subchondral bone subjected to alterations in microstructure in aging. We observed a larger volume and a greater thickness of the bone (Figure 4b). Then, we analyzed the cortical region of two tibia by μ‐CT. We were able to observe a higher bone volume and mineral density in the induced mice without any difference in bone thickness nor in its surface degradation, suggesting a lower porosity in the treated mice compared to the controls (Figure 4c, Figure S4b).

Collectively, these results demonstrated for the first time that a single short induction of cell reprogramming factors early in life positively regulates aging features of bone by protecting from osteoarthritis and osteoporosis in later life.

### 2.5 Amelioration of tissue structure and function at the onset of age‐related pathologies by an epigenetically related mechanism

Previous experiments demonstrated that a cyclic induction of OSKM 2 days a week, over the entire lifetime of a homozygous accelerated aging mice model, increased longevity, associated with a potential chronical epigenetic remodeling of histone marks (Ocampo et al., 2016). Thus, we decided to investigate whether our induction protocol of 2.5 weeks might engrave more permanently epigenetic marks that could explain the maintenance of tissues integrity we observed in aging, by preventing age‐related epigenetic alterations.

Among the various molecular changes involved in aging, an epigenetic drift revealed by DNA methylation has been associated with aging progression in mammalian (Pal & Tyler, 2016) and DNA methylation at specific CpG sites has turned out to be rather linked to biological aging than chronological aging (Field et al., 2018; Horvath & Raj, 2018). Interestingly, examination of aging‐associated DNA methylation has revealed that rejuvenation of this aging trait is initiated at early stages of the reprogramming process and is propagated during cell reprogramming (Gill et al., 2021; Olova et al., 2019), suggesting that the epigenetic rejuvenation might be dissociated from dedifferentiation, both mechanisms triggered by the reprogramming process.

Recently, Illumina released an array covering 285,000 CpG sites, allowing us to investigate for differentially methylated sites (DMS) across the mice genome. Thus, to investigate whether our treatment might prevent aging deteriorations by involving an epigenetic reprogramming, we first analyzed the extent of age‐related epigenetic modifications, comparing CpG DNA methylation of 8 months aged mice with 2‐month‐old mice in the various previously selected organs: kidney, spleen, lung, skin, liver, and heart. We identified DMS modified by aging (aging DMS), in each organ, as organ‐specific epigenetic signatures (Figure S5), and focused on these subsets for further analysis. We firstly demonstrate that our short reprogramming protocol of 2.5 weeks triggers immediate DNA methylation modifications, (hyper or hypomethylation), in each organ (Figure S6), suggesting that the distal positive effect observed on tissue deterioration in late age might be related to this early epigenetic reprogramming induced by OSKM.

**FIGURE 5:**
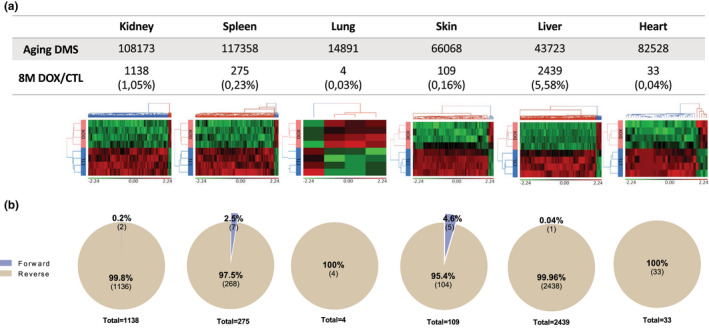
Differential methylation analysis reveals tissue‐specific modified DMS by a single short OSKM induction early in life, counteracting partially their aging drift. (a) Supervised hierarchical clustering of differentially methylated CpG loci between doxycycline‐treated (DOX, *n* = 4) and control mice (CTL, *n* = 4) in the 6 selected tissues at 8 months among aging DMS. Loci methylation levels of each CpG are represented by M value (log2 converted β values) which are shifted to mean of zero and scaled to standard deviation of one. Red and green colors represent, respectively, hypermethylated and hypomethylated CpG Loci. Doxycycline and control mice are, respectively, colored in red and blue. Significance level of differences between control and doxycycline‐treated mice was set at *p* < 0.005 according to one‐way ANOVA test. (b) Proportion of forward and reverse DMS between doxycycline‐treated and control mice among aging DMS. Forward and reverse DMS defined, respectively, whether methylation levels evaluated at 8 months in Doxycycline mice are modified in a similar or opposite way compared to aging methylation process.

To further analyze whether this early epigenetic reprogramming might prevent the epigenetic drift associated with aging, we selected among organ‐specific aging DMS, DMS between DOX and CTL mice at 8 months as the result of our short 2.5 weeks OSKM induction at 2 months. This results in the selection of a restricted set of DMS at 8 months per organ (Figure 5a), with very few sites shared by the different organs (Figure S7). Strikingly, by comparing the methylation status of each individual CpG site in DOX treated and CTL mice, for each organ, we found that the vast majority of the identified DMS at 8 months exhibit a reverse methylation status that counteracts the hyper or hypomethylation due to the age in all organs (Figure 5b, Figure S8), contrary to recently published results using a similar but not identical mouse model (Abad et al., 2013; Chondronasiou et al., 2022; Mosteiro et al., 2016). PCA of aging‐associated DMS illustrate the preventing effect of the epigenetic reprogramming triggered at 2 months by our single short induction protocol on the different organs (Figure S9).

Interestingly, as presented in Figure S10, looking at the distribution of DMS in the different gene regulatory elements, those organ‐specific DMS were mainly distributed in promoter regions suggesting that gene expression and/or regulatory pathways are likely modulated in the late ages as a consequence of the short OSKM induction at 2 months.

Based on these results, we compared DMS impacted by the treatment at 8 months with those impacted at 2 months among the aging DMS. Strikingly, very few CpG differentially methylated at 8 months impacted by the aging corresponds to those modified by OSKM at 2 months immediately after the treatment, whatever the organ (Figure S11). This indicates that the epigenetic reprogramming initiated at 2 months by our short induction protocol is not maintained, but is rather propagated during aging progression. This result is different from recently published results, revealing that short‐term partial reprogramming results only in minor epigenetic DNA methylation modification and in very few organs (Browder et al., 2022; Chondronasiou et al., 2022). Consistently, the DNA methylation clocks developed revealed no reduction of the calculated epigenetic age in the 1‐month short‐term partially reprogrammed animals (Browder et al., 2022). Only 7–10 months long‐term partially reprogrammed animals present a reduction of the calculated epigenetic age in the kidney and skin, but not in other tissues (Browder et al., 2022). Similarly, in the second study, using a different mouse model, a short OSKM induction of 1 week led to methylation changes in aging‐DM regions but very few in the spleen and liver with no histological changes associated (Chondronasiou et al., 2022).

Although gene expression is not directly correlated to DNA methylation changes, we found that GO analysis performed with the genes and their regulatory elements associated with the best DMS sets identified for each specific organ, revealed pathways with the ability to ameliorate the aging hallmarks (Figure S12) and to maintain the tissue homeostasis or to improve of the organ physiology (Figure S13). However, immunoblot analysis demonstrated that contrary to previously described results obtained by the chronic treatment of 1 mg/ml 2 days a week on homozygous progeria mice (Ocampo et al., 2016), proteins involved in senescence (p53, p21, p16), as well as histone marks (H4K20me3, H3K9me3, H3K27me3) described to be ameliorated, did not show any significant differences at 8 months in the various organs studied when a single short OSKM induction is applied early in life (Figure S14). Therefore, the amelioration of tissue deterioration induced by our short induction protocol applied early in life likely involves different molecular mechanisms linked to the persistence of OSKM expression during 2.5 weeks.

Altogether, our results are consistent with an epigenetic reprogramming whose propagation might be involved in the maintenance of tissue ultrastructure and the amelioration of organs physiology in aging, preventing age‐related diseases.

## 3 CONCLUSION

In this paper, we developed an intermediate model, between wild‐type and severely affected homozygotes, to study rejuvenating interventions. The heterozygotes live about 35 weeks compared to 20 weeks for homozygotes and 2 years for wild type. Ocampo and collaborators developed a specific induction protocol showing that by inducing a short cyclic and chronic induction of the Yamanaka reprogramming factors OSKM through the entire life of an accelerated‐aging mouse model, they extended the lifespan of mice and improved some age‐related hallmarks (Ocampo et al., 2016). We thus decided to explore the impact of reprogramming on aging and age‐related pathologies in longer‐living animals and to further manipulate the experimental conditions for the induction of the OSKM factors.

Consequently, we simplify and refine the Ocampo protocol using heterozygous progeric and non‐progeric mice to evaluate the effect on aging and we reveal for the first time that even a single short treatment can improve long‐term outlook. Strikingly, we discovered that a single step of cellular reprogramming at the level of the organism by a 2.5 weeks treatment on 2 month‐young heterozygotes improves body composition lifelong and impacts both lifespan and healthspan, protecting tissues and organs that deteriorate during aging. Although we cannot define precisely the mechanism involved, this indisputable “distal” effect is associated with an epigenetic reprogramming propagated in aging and finally associated with genes pathways with the ability to alleviate aging hallmarks and to improve tissue‐specific physiology. Although the molecular mechanisms involved in this epigenetic propagation have to be further analyzed, our short reprogramming protocol is able to ultimately counteract some CpG modifications associated with aging. We conclude that our unique OSKM induction protocol is involved in the initiation and propagation of a favorable epigenetic reprogramming finally leading to an improvement of cell and tissues physiology in old age.

Altogether, our results open the door to potential clinical applications based on a safe reprogramming strategy for therapeutic intervention to prevent tissue aging deterioration and age‐related pathologies.

## 4 METHODS

### 4.1 Mice model and housing

Experiments were performed on heterozygous progeric mice R26^rtTA/+^;Col1A1^4F2A/+^;Lmna^G609G/+^ generated by the crossing of two lines of the following genotypes: Reprogramming mice from JAX (STOCK Gt(ROSA)26Sortm1(rtTA+M2)Jae Col1a1tm3(tetO‐Pou5f1,‐Sox2,‐Kfl4,‐Myc)Jae/J (JAX: 011004)) in homozygous form for the two transgenes (two copies for the rtTA transactivator (R26rtTA/rtTA) and two copies for the 4 reprogramming factors (OSKM) cassette (Col1A14F2A/4F2A) and progeria mice LmnaG609G/+ (MGI: 5295747)) (Osorio et al., 2011), recapitulating the human HGPS accelerated aging phenotype through the accumulation of the prelamin A truncated form, also called progerin.

The Project was validated by the Ethical committee (Agreement APAFIS #21760), and animal care and use were performed in accordance with the recommendations of the European community (2010/63/UE).

### 4.2 Longevity studies

To induce reprogramming in our animals, we implemented either lifelong or short induction protocols. For both, doxycycline was administered in drinking water in opaque bottles. All protocols started at the age of 2 months and were carried out on animals of genotype R26^rtTA/+^;Col1a1^4F2A/+^;Lmna^G609G/+^. Two lifelong protocols were used and lasted until the animals died. The first one consists of a cyclic induction 2 days a week at 1 mg/mL of doxycyline. The second that we have developed consists of a lower induction at 0.2 mg/ml of doxycycline. In addition, two short induction protocols were developed. Both consists in inducing animals for only 2.5 weeks at the age of 2 months, at 0.2 mg/ml of doxycycline for the first and 0.5 mg/ml for the second. The last one was also developed on non‐progeric animals of genotype R26^rtTA/+^;Col1a1^4F2A/+^;Lmna^+/+^.

### 4.3 Body composition analysis

Mice whole‐body composition (fat and lean masses) was measured every month throughout the study by quantitative magnetic resonance with a whole‐body composition analyzer (EchoMRI™700 Echo Medical Systems, Houston, TX, USA) according to the manufacturer's instructions. Each animal was individually weighted before each measurement.

### 4.4 Functional assays

We measured the motor coordination of mice, on a rotarod machine with automatic timers and falling sensors (47650 Rota‐Rod, Ugo Basile®), set up in a ramp mode from 5 rpm to 40 rpm over 300 s. The latency to falling is recorded. Each animal ran 3 times for a maximal time of 10 min followed by 20 min of resting between each run once a month until time of death.

Muscular strength of mouse front legs was performed by a vertical grip strength test (Bio‐GS3, BioSeb). Mice were held by the tail, placed above the gripping bar until they grasped the bar, and pulled up until grip was released. Maximal grip force developed was measured 3 times, with a few minutes rest, in its cage between each test. A test was performed once a month until time of death.

### 4.5 Measurement of histopathological parameters

For the thickness measurement of the various skin layers, images with 20x digital magnification were taken on a minimum of 3 identically oriented skin sections per individual. Hundreds of measurements were then carried out for each layer of the skin.

For spleen, the architecture of the marginal zone was evaluated according to the scoring method developed previously for evaluation of age‐related alteration in mice (Birjandi et al., 2011), based on percentage of distortions between the white pulp and the marginal zone. The characterization of the alteration ranges from minimal (score between 7 and 8) to severe (score between 0 and 2).

For kidney, the mesangial area around the renal glomeruli was analyzed using ImageJ software (NIH).

### 4.6 Tissue fibrosis

Fibrosis levels were quantified after MT staining in lungs or SR staining in spleen, kidneys, liver, and heart. A color deconvolution procedure was applied for each, using the appropriate function available on Fiji software, based on the MRI developed plugin (http://dev.mri.cnrs.fr/projects/imagej‐macros/wiki/Fibrosis_Tool). Then, channels of interest were selected, and the background noise was subtracted using the MaxEnthropy auto‐threshold method.

The areas covered by fibrosis were then measured and quantified.

### 4.7 Cartilage degradation and bone structure evaluation

A Leica Microsystems TCS SP5‐II confocal laser scanning microscope was used to acquire images of the articular cartilage of the lateral and median plateau. The cartilage was scanned in‐depth (XYZ mode) using the following parameters (voxel size 6 μm, 5x dry objective, and UV laser light source at 405 nm). Image stacks were used to reconstruct a 3D image of the cartilage as well as for quantification.

Osteoporosis and surface degradation were measured on the cortical and subchondral region (lateral and median plateau in the knee joint) of the tibia. The samples were scanned by X‐ray microtomography (μ‐CT) on a SkyScan 1176 scanner (Bruker) using CTAn software (Bruker) and the following parameters (aluminum filter, 45 kV, 500 μA, resolution of 18 μm, 0.5° rotation angle). Scans were then reconstructed using NRecon software (Bruker). The 3D images of the joints were reconstructed using Avizo software (Avizo Lite 9.3.0, FEI Visualization Sciences Group).

For the μ‐CT and confocal laser scanning microscopy experiments, each sample was independent and represented an experimental unit providing a unique result.

### 4.8 DNA methylation studies

For DNA methylation studies, genomic DNA was extracted from lysed tissues using Invitrogen TRIzol™ reagent experimental protocol for DNA isolation (Catalog Numbers 15596026) and further processed by Life & Brain GmbH Platform Genomics for DNA methylation profiling on “Illumina Infinium Mouse Methylation arrays” according to the manufacturer's instructions. For each CpG locus, normalized methylation levels (β values), ranging from 0 (completely unmethylated) to 1 (completely methylated), were calculated and used for differential methylation analysis between mice group (96 mice: 4 CTL and 4 DOX in 6 tissues at 2 months and 6 months). One‐way‐ANOVA statistical test was performed on log2 converted β values (M value = log2(β/[1‐β])). The significant differentially methylated CpG loci (*p* < 0.05) were used in hierarchical clustering and GO analysis.

Methylation analysis was conducted using Partek® Genomics Suite® software.

## AUTHOR CONTRIBUTIONS

Conceptualization and Supervision: J.M.L., O.M. Funding acquisition and project administration: J.M.L. Data analysis: Q.A., E.L.B., P.B., O.M., J.M.L. Writing original draft: Q.A., E.L.B., P.B., O.M., J.M.L. Animal experiments and sample analysis: Q.A., E.L.B., C.L., O.M. Histology: Q.A. Methylation analysis: P.B., E.L.B. Animal model: C.L. Bone and cartilage: Q.A., K.T., D.N., C.J. Animal metabolism profiling: E.L.B., C.B.G., L.P., F.C. Scientific and technical support: N.B., M.G., F.E., J.V., C.H.

## FUNDING INFORMATION

Research was supported by the University of Montpellier, the CHRU Montpellier and Grant from Dotation INSERM, Ligue Nationale Contre le Cancer “Equipe Labellisée (2015–2019) and from la Ligue Comité régional de l'Hérault (2020–2021).

## CONFLICT OF INTEREST

The authors declare that they have no competing interests.

## Supporting information


Text S1

Figure S1

Figure S2a

Figure S2b

Figure S3

Figure S4

Figure S5

Figure S6

Figure S7

Figure S8

Figure S9

Figure S10

Figure S11

Figure S12

Figure S13

Figure S14

## Data Availability

The datasets generated and analyzed during the current study are available from the corresponding authors on reasonable request.
