# Supplementary material for: A single short reprogramming early in life initiates and propagates an epigenetically related mechanism improving fitness and promoting an increased healthy lifespan
Source: Aging Cell. 2022 Oct 17;21(11):e13714. doi: 10.1111/acel.13714 (PMC9649606; doi:10.1111/acel.13714)
Supplement: Supplementary file 1 — Text S1 [file ACEL-21-e13714-s005.pdf]

# A single short reprogramming early in life initiates and propagates an epigenetically related mechanism improving fitness and promoting an increased healthy lifespan

Quentin Alle[1†], Enora Le Borgne[1†], Paul Bensadoun[1‡], Camille Lemey[1‡], Nelly Béchir[1], Mélissa Gabanou[1], Fanny Estermann[1], Christelle Bertrand-Gaday[3,7], Laurence Pessemesse[3], Karine Toupet[1,4], Romain Desprat[5], Jérôme Vialaret[1,6], Christophe Hirtz[1,6], Danièle Noël[1,4], Christian Jorgensen[1,4], François Casas[3,7], Ollivier Milhavet[2,5]* and Jean-Marc Lemaitre[1,5]*

[1] *IRMB, Univ Montpellier, INSERM, Montpellier, France.*

[2] *IRMB, Univ Montpellier, INSERM, CNRS, Montpellier, France.*

[3] *DMEM, Univ Montpellier, INRAE, Montpellier, France.*

[4] *ECELLFrance Montpellier Facility, Univ Montpellier, France*

[5] *SAFE-iPSC Facility, CHU Montpellier, Montpellier, France*

[6] *PPC Facility, CHU Montpellier, Montpellier, France*

[7] *RAM-METAMUS, Univ Montpellier, INRAE, Montpellier, France*

[†]First authors contribute equally to this work
[‡]Second authors contribute equally to this work

*Corresponding authors: Jean-Marc Lemaitre (jean-marc.lemaitre@inserm.fr) and Ollivier Milhavet (ollivier.milhavet@inserm.fr)

**SUPPLEMENTARY INFORMATIONS**

## Supplementary extended Methods:

### Mice model and housing

Mice allowing *in vivo* transient reprogramming were originally developed by Rudolf Jaenisch (Whitehead Institute, Massachusetts Institute of Technology, USA) and purchased from Jackson Laboratories Laboratories (STOCK Gt(ROSA)26Sor$^{tm1(rtTA+M2)Jae}$ Col1a1$^{tm3(tetO-Pou5f1,-Sox2,-Kfl4,-Myc)Jae}$/J (JAX: 011004). These mice are bred in homozygous form for the two transgenes (two copies for the rtTA transactivator (R26$^{rtTA/rtTA}$) and two copies for the 4 reprogramming factors (OSKM) cassette (Col1A1$^{4F2A/4F2A}$). The murine line exhibiting the accelerated aging phenotype (Hutchinson-Gilford progeria syndrome) was originally developed by Carlos Lopez-Otin from University of Oviedo, Spain (Lmna$^{tm1.1Otin}$ (MGI: 5295747))(Osorio et al. 2011). This line carries the G609G mutation on the LMNA gene, leading to the activation of a cryptic splicing site and the accumulation of the prelamin A truncated form, also called progerin. This line is bred in heterozygous form for the progeria mutation (Lmna$^{G609G/+}$).

Experimental groups were generated by the crossing of these two lines and are represented by the following genotype: progeric R26$^{rtTA/+}$;Col1A1$^{4F2A/+}$;Lmna$^{G609G/+}$ . Animal care and use for this study were performed in accordance with the recommendations of the European community (2010/63/UE). The Project was validated by the Ethical committee of the French Ministry of Research through the agreement APAFIS #21760. All mice were produced at PCEA (Agreement n°A34-172-45) and transferred to the analysis platform one week before starting experiments for habituation. The procedures and protocols concerning body composition and functional analysis were performed on the Metamus platform, at the DMEM unit of the INRAE-UM in Montpellier, France (Veterinary Services National Agreement n° E34-172-10, 04 March 2019). All the others

procedures and protocols were performed at INM (Agreement n° C34-172-36). Mice were housed in groups in filter-top cages with free access to standard diet (A04, SAFE diets, Augy, France) and tap water. They were maintained in a temperature-controlled room (24°C ±1°C) in a standard 12:12 light-dark cycle (lights at 7:30 am). All cages were enriched with nesting materials (cellulose squares, SAFE). Animal behavior was checked daily for welfare and health status and mice weight was monitored every two weeks. For mice subjected to body composition analysis and functional assays, cages were also enriched with a hanging red polycarbonate tunnel. This tube is equivalent to the animal holder used for EchoMRI measures and allows mice habituation to a restrained environment.

**Tissue sampling and preparation**

In these experiments, animals of the short-term OSKM induction group (0.5 mg/ml of doxycycline at 2 months for 2.5 weeks and untreated controls) were sacrificed either just after the treatment of at 8 months following an ethical and authorized procedure, 6 months after the end of the induction. Nine controls animals and eight treated animals with both sexes were used in these studies.

Organs were removed and dissected according to RITA standardization procedures (Morawietz et al. 2004). The samples intended for tissue structure analysis were fixed in 4% PFA, for 24 h at room temperature, then washed three times in 1X PBS and stored in 70% EtOH at 4°C before staining.

At the same time, tissue samples were also snap-frozen in liquid nitrogen for protein and RNA content analysis. The samples were placed in 1.5 ml tubes with stainless steel beads of different sizes (1 x 3mm, 2 x 2mm and 4 x 1mm in diameter) and ground in an adequate buffer using the Qiagen MixerMill MM300 tissue lyser. More details are available in dedicated sections.

For cartilage and bone analysis, whole hind legs were fixed in 4% PFA for 7 days at room temperature, washed three times with 1X PBS and stored in 70% EtOH at 4°C. The soft tissues were removed manually before analysis.

**Histological staining**

Stainings were carried out by the Montpellier Experimental Histology Network platform (RHEM) using standardized procedure based on Leica Autostainer XL technology. For each organ sample, 3 µm of thickness slices were produced and mounted on slides. For each animal sampled, Hematoxylin, Eosin and Saffron (HES) staining was performed to assess the of tissue's structural parameters. Sirius Red (SR) and Masson's Trichrome (MT) staining were also used to measure the fibrosis level in those tissues. All slides were scanned by the Montpellier Ressources Imagerie platform (MRI), using a Hamamatsu Photonics NanoZoomer, fitted with a dry x40 objective. NDP.view2 software (Hamamatsu) was used for viewing virtual scans for image acquisition. For all the experiments, acquisition and display settings were identical between each sample to guarantee a reliable quantification.

**Statistical analyses**

For the µ-CT and confocal laser scanning microscopy experiments, each sample was independent and represented an experimental unit providing a unique result.

Statistical analyzes were performed with Prism 7 software (GraphPad). In all histograms, data are presented as the mean ± SEM. For the comparisons of two groups, an unpaired (*in vivo*) or paired (*in vitro*) t-test was used. For the comparisons of the survival curves a log-rank test (Mantel-Cox) was used. For the hair recovery experiment a Chi-square test was used. A value of $p<0.05$ was considered significant.

**DNA methylation**

Genomic DNA (gDNA) was extracted from animal lysed tissues using Invitrogen TRIzol™ reagent experimental protocol for DNA isolation (Catalog Numbers 15596026) enabling to isolate sequentially RNA, DNA and proteins from the same sample. gDNA was further processed by Life &

Brain GmbH Platform Genomics for DNA methylation profiling. Briefly, bisulfite conversion was performed on qualified gDNA and DNA Methylation levels were measured on "Illumina Infinium Mouse Methylation arrays" according to the manufacturer's instructions. This new array quantitatively targets over 285 000 CpGs sites across the mice genome. Methylation and Gene Ontology (GO) analysis were conducted using Partek® Genomics Suite® software. We used Illumina's standard normalization with filtering option to exclude probe on sexual chromosomes. For each CpG locus, normalized methylation levels (β values), ranging from 0 (completely unmethylated) to 1 (completely methylated) were calculated and used for differential methylation analysis between mice group (96 mice: 4 CTL and 4 DOX in 6 tissues at 2 months and 8 months).

A one-way-ANOVA statistical test was performed on log2 converted β values (M value = $\log_2(\beta/(1-\beta))$ to evaluate the significance of differentially methylated CpG loci. The significant differentially methylated CpG loci ($p<0.05$) were used in hierarchical clustering and GO analysis.

Principal Component analysis (PCA) were applied on normalized data set of the 6 revealed tissue specific aging related signatures by comparing 8 month treated mice (8M DOX, n=4), 8 months non treated mice (8M CTL, n=4) and 2 months non treated mice (2M CTL, n=4). This exploratory analysis enables us to visualize long term rejuvenation effect of the treatment applied early in life. Methylation analysis was conducted using Partek® Genomics Suite® software.

**Gene expression**

After Trizol extraction, assay and quality control of the RNA, a cDNA retrotranscription step is performed using the Maxima First Strand cDNA Synthesis Kit (#K1672, Thermo Scientific) with DNase pre-treatment to eliminate possible genomic DNA contaminants. Analysis of transcript expression levels were performed using 96.96 Dynamic Array IFC read on Biomark™ HD Reader after a sample pre-amplification step.

**Dot blot**

Frozen tissue samples were ground in 1.5mL Eppendorfs using the Qiagen MixerMill MM300 tissue lyser and steel beads (1 x 3mm, 2 x 2mm and 4 x 1mm in diameter). Tissue lysis was performed in RIPA buffer (Fisher Scientific) supplemented with a protease and phosphatase inhibitors cocktail (Fisher Scientific). Samples were kept on ice during lysis and then centrifuged at 14,000 *xg* for 15 min at 4°C to remove cellular debris. Protein content of the lysates is then quantified by using the Pierce BCA protein assay kit (Fisher Scientific).

The non-denaturated samples were directly transferred to membrane by aspiration using a 96-well apparatus (Carl Roth T790.1) and each well was then rinsed twice with 1X TBST, and once with 1X TBS. Membranes were then blocked for 1 H with 1X TBS with 10 % non-fat dry milk. All antibody labeling was performed in 1X TBST buffer with 1% BSA. Primary antibody labelling was performed overnight at 4°C. The membranes were then washed 3 times with 1X TBST and secondary labeling was performed for 1H at room temperature. Membranes were washed twice with 1X TBST and then once with 1X TBS before revelation. The revelation was performed with the SuperSignal West Pico Chemiluminescence Substrate kit (Fisher Scientific) and using a ChemicDoc reader (Bio-Rad) and Image Lab 4.1 software.

Primary antibodies: Rabbit polyclonal anti-H3K27me3 antibody (Active Motif 39156, 39158; WB: 1/1000). Rabbit polyclonal anti-H3K9me3 antibody (Abcam ab8898; WB: 1/1000). Mouse monoclonal anti-H4K20me3 antibody (Active Motif 39671, 39672; WB: 1/1000). Mouse monoclonal anti-p53 antibody (Cellsignaling #2524; WB: 1/1000). Mouse monoclonal anti-CDKN2A/p16INK4a antibody (Abcam ab54210; WB: 1/1000). Mouse monoclonal anti-p21 antibody (Santa-Cruz Biotechnology, Inc. sc-6246 clone F-5; WB: 1/200). Rabbit polyclonal anti-H3 antibody (Abcam ab 1791; WB: 1/5000).

Secondary antibodies: Peroxidase-conjugated goat anti-mouse IgG antibody (Jackson ImmunoResearch; AffiniPure 115-035-146; WB: 1/50000). Peroxidase-conjugated goat anti-rabbit IgG antibody (Jackson ImmunoResearch; AffiniPure 111-035-144; WB: 1/50000).

**Supplementary figure legends:**

**Supplementary Figure 1: A single short transient OSKM induction applied early in life, increases late age lifespan in non progeric mice.**

Scheme of short-term induction protocol performed on non progeric mice $R26^{rtTA/+}$;$Col1a1^{4F2A/+}$;$Lmna^{+/+}$ performed by administrating doxycycline in the drinking water for 2.5 weeks starting at 2 months old at 0.5 mg/ml (green curve). Survival curves of short-term doxycycline treated mice after induction compared to untreated mice (grey curve) with the same genotype are presented. Statistical analysis of curves was performed at the corresponding indicated percent survival. Median survival for the 3rd quartile and maximum lifespan value is presented. * p=0.0227 according to log-rank (Mantel-Cox) test.

**Supplementary Figure 2: OSKM Gene expression in various tissues.**

A) QPCR analysis at different time points of OCT4 and SOX2 mRNA levels in indicated organs of R26rtTA/+; Col1a14F2A/+; LmnaG609G/+ progeric mice induced (DOX) or not (CTL) at 2 months during 2.5 weeks by administration of Doxycycline at 0.5 mg/mL. Skin fibroblasts collected from R26rtTA/+; Col1a14F2A/+;LmnaG609G/+ progeric mice were induced by doxycycline 1 µg/ml during 4 days in vitro and used as positive control. (A) Tissues studied are wat (WAT), muscle (MUSCLE) and skin fibroblasts (FIBROS) at different time points. (B) Tissues studied are SKIN, KIDNEY, SPLEEN, LIVER, LUNG and HEART at different time point. Data are expressed as mean ± SEM fold change of indicated transcript level in treated vs control animals. Significance of differences in gene expression are indicated (one-way ANOVA test except for heart where unpaired t-Student's test was applied). (#) Important basal expression of Sox2 in lungs did mask specific expression of Sox2 from the transgene. A value of $p<0.05$ was considered significant and presented as, **** $p<0.0001$; *** $p<0.001$; ** $p<0.01$; * $p<0.05$.

**Supplementary Figure 3: Tissue structure and age-related tissue fibrosis is improved in aging by a single OSKM induction early in life.**

Histological analyzes of liver **(A)** and heart **(B)** sections were performed after staining with Hematoxylin, Eosin and Saffron (HES, on left panels) and fibrosis after Red Sirius (RS, on right panels). **(A)** Morphologic illustration of liver tissue structure and fibrosis analysis in treated and untreated mice. **(B)** Morphologic illustration of heart left ventricle tissue structure and fibrosis analysis in treated and untreated mice. All scales represent 150µm. All tissues were analyzed on 8 months old $R26^{rtTA/+}$;$Col1a1^{4F2A/+}$;$Lmna^{G609G/+}$ progeric mice. CTL represents untreated mice and DOX represents treated mice with 0.5 mg/ml doxycycline during 2.5 weeks at the age of 2 months following short-induction protocol. All Measurements of areas and distances were performed on ImageJ software.

**Supplementary Figure 4: A single short OSKM induction, early in life, prevents osteoarthritis and osteoporosis in age mice.**

**(A)** Representative 3D reconstruction of sub-chondral bone. Rough appearance is representative of bone degradation. Sub-chondral bone surface degradation was measured in the lateral and medial plateau. **(B)** µ-CT histomorphometric analysis of tibias cortical region (blue box and arrow). Cortical bone thickness and surface degradation were measured on both tibias. Bone tissues were analyzed on 8 months old *$R26^{rtTA/+}$*; *$Col1a1^{4F2A/+}$*;$Lmna^{G609G/+}$ progeric mice. CTL represents untreated mice and DOX represents treated mice with 0.5 mg/mL doxycycline during 2.5 weeks at the age of 2 months following short-induction protocol. * $p<0.05$ according to unpaired t-test, one-tailed, with Welch's correction.

**Supplementary Figure 5: Differentially methylated CpG related to aging (aging DMS), in the different organs.**

Supervised hierarchical clustering of differentially methylated CpG loci between untreated mice 2 months (n=4) and 8 months (n=4) in the 6 selected tissues. Loci methylation levels of each CpG are represented by M value (log2 converted β values) which are shifted to mean of zero and scaled to standard deviation of one. Red and green color represents respectively hypermethylated and hypomethylated CpG Loci. 8 month and 2 months aged mice are respectively colored in orange and green. Significance of differences between mice group are indicated (one-way ANOVA test).

Each Hierarchical clustering were made on the 10 000 most significant CpG loci.

**Supplementary Figure 6: Differentially methylated CpG, among the aging DMS, modified by the short induction protocol at 2 months, in the different organs.**

Supervised hierarchical clustering of differentially methylated CpG loci between doxycycline treated (DOX, n=4) and control mice (CTL, n=4) in the 6 selected tissues at 2 months. Loci methylation levels of each CpG are represented by M value (log2 converted β values) which are shifted to mean of zero and scaled to standard deviation of one. Red and

green color represents respectively hypermethylated and hypomethylated CpG Loci. Doxycycline and control mice are respectively colored in red and blue.

Significance of differences between control and doxycycline treated mice are indicated (one-way ANOVA test).

**Supplementary Figure 7: Differentially methylated CpG, among the aging DMS, modified by the short induction protocol at 8 months, in common in the different organs.**

Upset plot representing the intersections between sets of DMS by organ comparing doxycycline treated mice and control mice at 8 months among aging selected DMS.

**Supplementary Figure 8: Example of forward and reverse differentially methylated CpG, among the aging DMS by organ.**

Representative examples for all organs of DMS that are either hypermethylated with aging and reversely hypomethylated in treated mice at 8 months (kidney, skin, liver and heart) or hypomethylated with aging and reversely hypermethylated in treated mice at 8 months (spleen and lung).

**Supplementary Figure 9: PCA representation of organ specific aging-associated DMS at 8 months reveal the preventing effect of the epigenetic reprogramming triggered by a single short OSKM induction early in life on aging epigenetic drift observed in old mice.**

Principal Component Analysis of differentially methylated CpG loci between 8 months' doxycycline treated (DOX, n=4) and 8 months' control mice (CTL, n=4) in the 6 selected tissues among the tissue specific aging DMS. Doxycycline and control mice are respectively colored in red and blue. 2 and 8 months-old mice are represented by a triangle and square respectively. Revealed clusters of each group are marked in color.

**Supplementary Figure 10: Distribution of the differentially methylated CpG, at 8 months, in the different gene regulatory elements in the different organs.**

6 gene regulatory elements were analyzed: promoters, coding DNA sequences (CDS), intronic regions (intron), non-coding sequences (non-coding), 5' and 3' untranslated regions (UTR). The distribution of DMS among them was plotted as a percentage of the total DMS identified for every organ at 8 months.

**Supplementary Figure 11: Comparison of Differentially methylated CpG modified by the short induction protocol at 2 months with the sites differentially methylated at 8 months, as a consequence of the treatment.**

Common DMS modified by the DOX treatment at 2 and 8 months among the selected aging DMS were compared for each studied organ and represented as individual Venn diagrams.

**Supplementary Figure 12: GO analysis for the nine hallmarks of aging mitigation with the genes associated to the differentially methylated loci, in the different organs at 8 months**

Gene ontology analysis was made to help the interpretation of genes associated to the differentially methylated loci, in the different tissues.

First, Gene set analysis was performed on significant (p<0,05) differentially methylated CpG loci between experimental mice control group (CTL, n=4) and doxycycline treated group (DOX, n=4) in each tissue. “Ensembl Transcripts release 100” of Mus musculus. GRCm38 was chosen as annotation database. Promoter region was configured as 5000 base pairs upstream and 3000 base pairs downstream from the transcription start site (TSS).

We used Partek gene ontology workflow recommended parameters (Fisher’s exact test, restrict analysis to functional groups with more than 2 genes and use the genes from genes ontology database as background)

Gene sets over represented on the differentially methylated CpG loci associated genes are reveals by an enrichment score greater than 1 (enrichment score = -ln(enrichment p-value).

To help the interpretation of theses age related DML, we then focused on GO term related to the famous 9 hallmarks of aging (telomere attrition, DNA Damage, mitochondrial dysfunction, senescence, stem cell exhaution, proteostasis, nutrient sensing, epigenetics, intercellular communication). For each hallmark, the 2 best GO term (best enrichment score and best number of genes) were plotted for each organ’s signature.

**Supplementary Figure 13: GO analysis for tissue specific organ homeostasis maintenance with the genes associated to the differentially methylated loci, in the different organs at 8 months**

Gene set analysis was performed on significant ($p<0,05$) differentially methylated CpG loci between experimental mice control group (CTL, n=4) and doxycycline treated group (DOX, n=4) in each tissue. "Ensembl Transcripts release 100" of Mus musculus. GRCm38 was chosen as annotation database. Promoter region was configured as 5000 base pairs upstream and 3000 base pairs downstream from the transcription start site (TSS).

We used Partek gene ontology workflow recommended parameters (Fisher's exact test, restrict analysis to functional groups with more than 2 genes and use the genes from genes ontology database as background)

We then focused on the tissue related GO term and plotted 18 GO term for each organ's signature (9 higher enrichment score and 9 higher number of genes found in the go term list).

**Supplementary Figure 14: Histone marks changes at 8 months and senescence as a consequence of 2.5 weeks the OSKM induction at 2 months.**

**(A)** Dot blot assay against histone marks H4K20me3, H3K9me3 and H3K27me3 performed on tissue extracts from progeric reprogrammable mice at 8-months-old after an early short transient reprogramming. Proteins are detected by immunostaining with specific antibodies. Histone 3 has been used as loading control. **(B)** Quantification of mRNA expression levels of p16, p21 and p53 by IFCs Dynamic Array performed on tissue extracts from progeric reprogrammable mice (*Col1a1*$^{4F2A/+}$ *R26*$^{rtTA/+}$ Lmna$^{G609G/+}$) at 8-months-old after an early short transient reprogramming (DOX 0.5 mg/mL for 16 days at 2-months-old). **(C)** Dot blot assay against senescence markers p16, p21 and p53 performed on tissue extracts from progeric reprogrammable mice at 8-months-old after an early

short transient reprogramming. Proteins are detected by immunostaining with specific antibodies. Histone 3 has been used as loading control.

## Supplementary References:


Morawietz G, Ruehl-Fehlert C, Kittel B, Bube A, Keane K, Halm S, Heuser A, Hellmann J. 2004. Revised guides for organ sampling and trimming in rats and mice--Part 3. A joint publication of the RITA and NACAD groups. *Experimental and toxicologic pathology : official journal of the Gesellschaft fur Toxikologische Pathologie* **55**: 433-449.

Osorio FG, Navarro CL, Cadiñanos J, López-Mejía IC, Quirós PM, Bartoli C, Rivera J, Tazi J, Guzmán G, Varela I et al. 2011. Splicing-directed therapy in a new mouse model of human accelerated aging. *Science translational medicine* **3**: 106ra107.
