# Supplementary figures and images for: A single short reprogramming early in life initiates and propagates an epigenetically related mechanism improving fitness and promoting an increased healthy lifespan
Source: Aging Cell. 2022 Oct 17;21(11):e13714. doi: 10.1111/acel.13714 (PMC9649606; doi:10.1111/acel.13714)

## Supplementary Figure 1

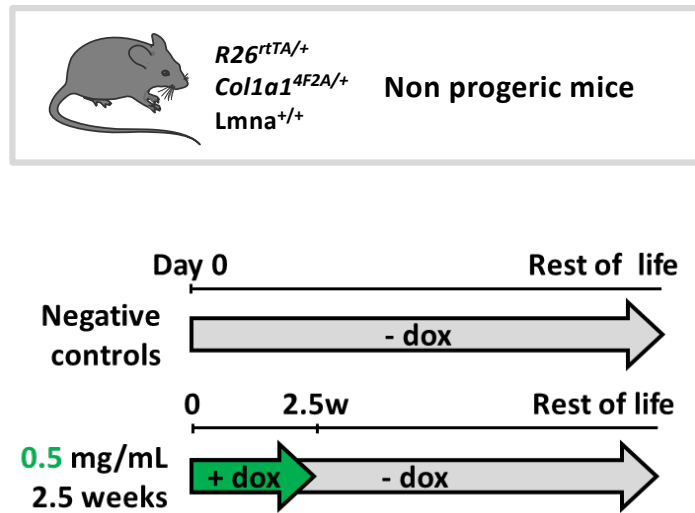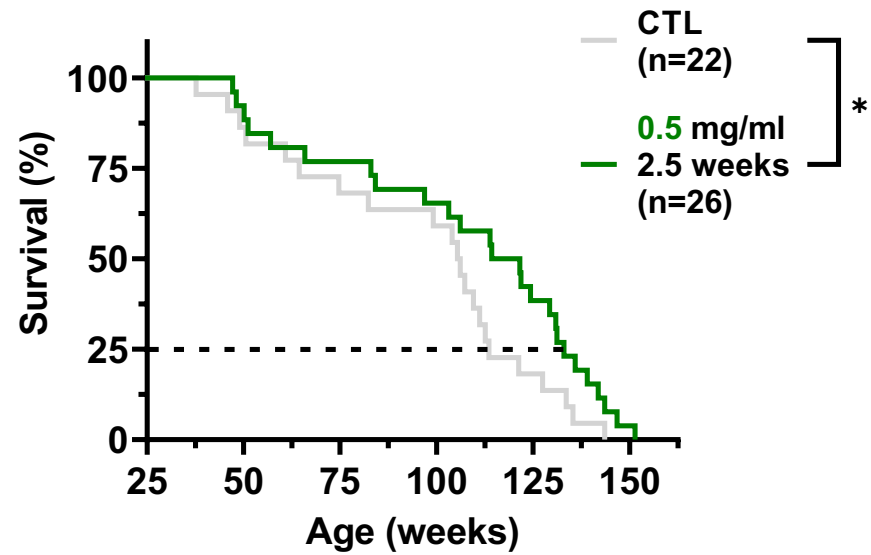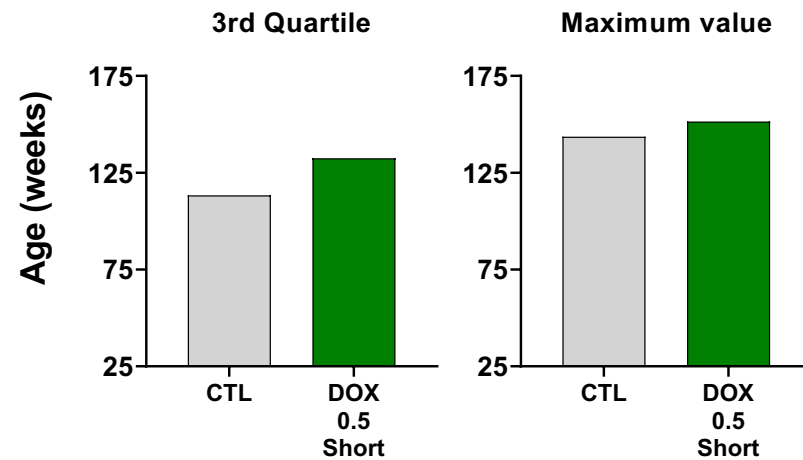

Supplement: Supplementary file 2 — Figure S1 [file ACEL-21-e13714-s002.pdf]

Supplementary Figure 2 A

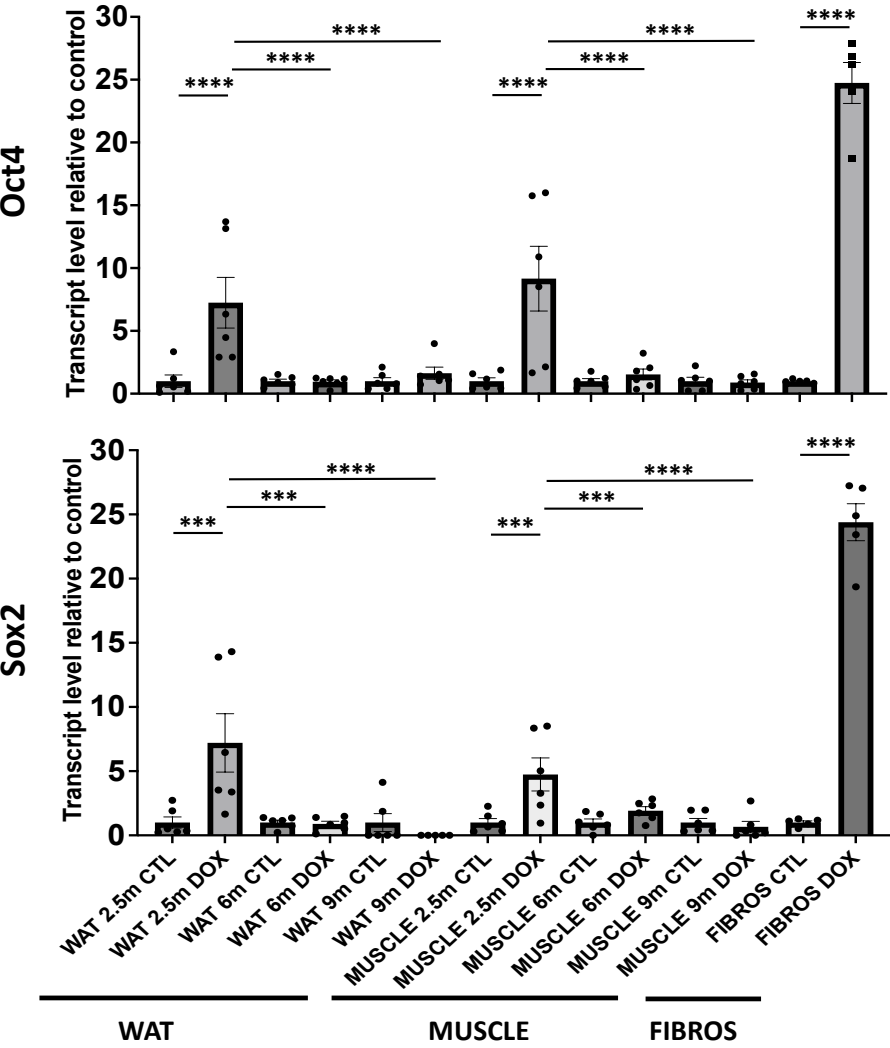

Supplement: Supplementary file 3 — Figure S2a [file ACEL-21-e13714-s012.pdf]

Supplementary Figure 2 B

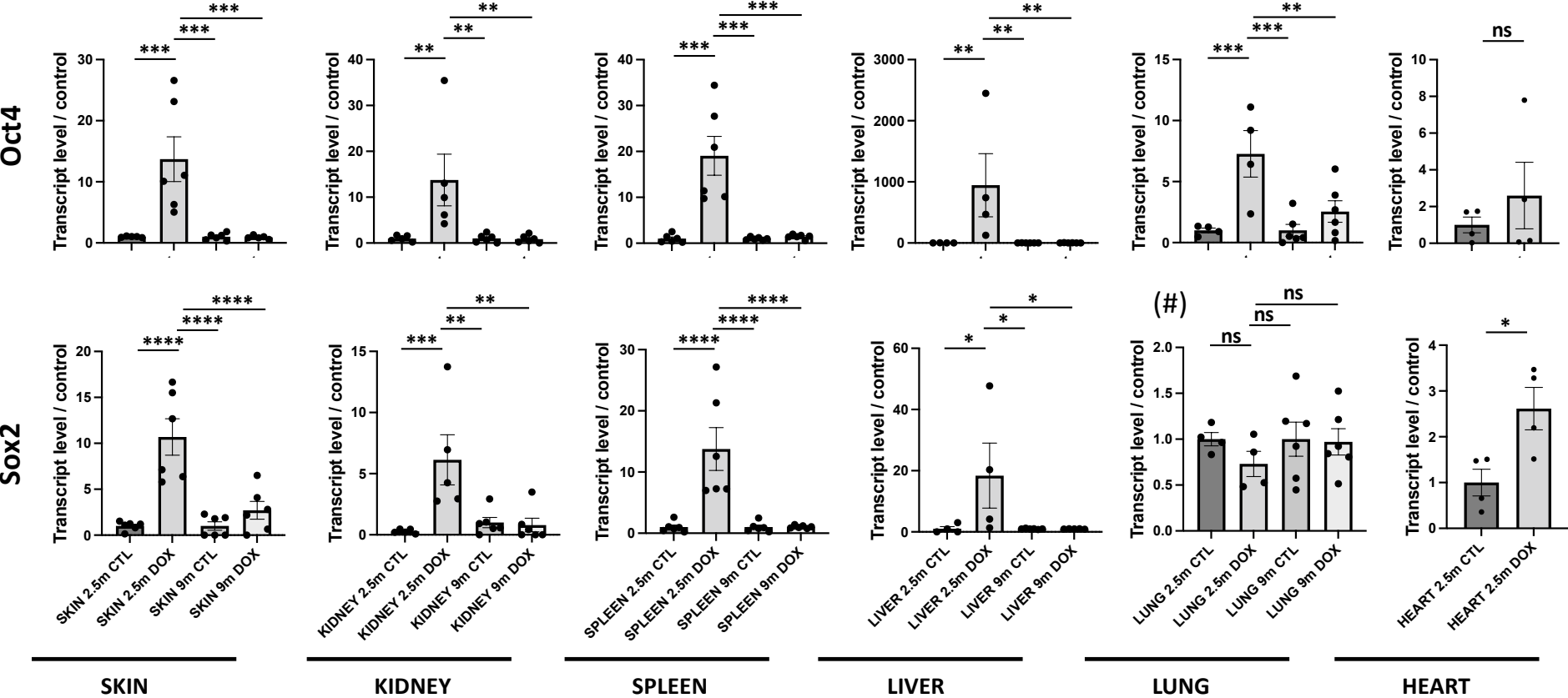

Supplement: Supplementary file 4 — Figure S2b [file ACEL-21-e13714-s010.pdf]

Supplementary Figure 3

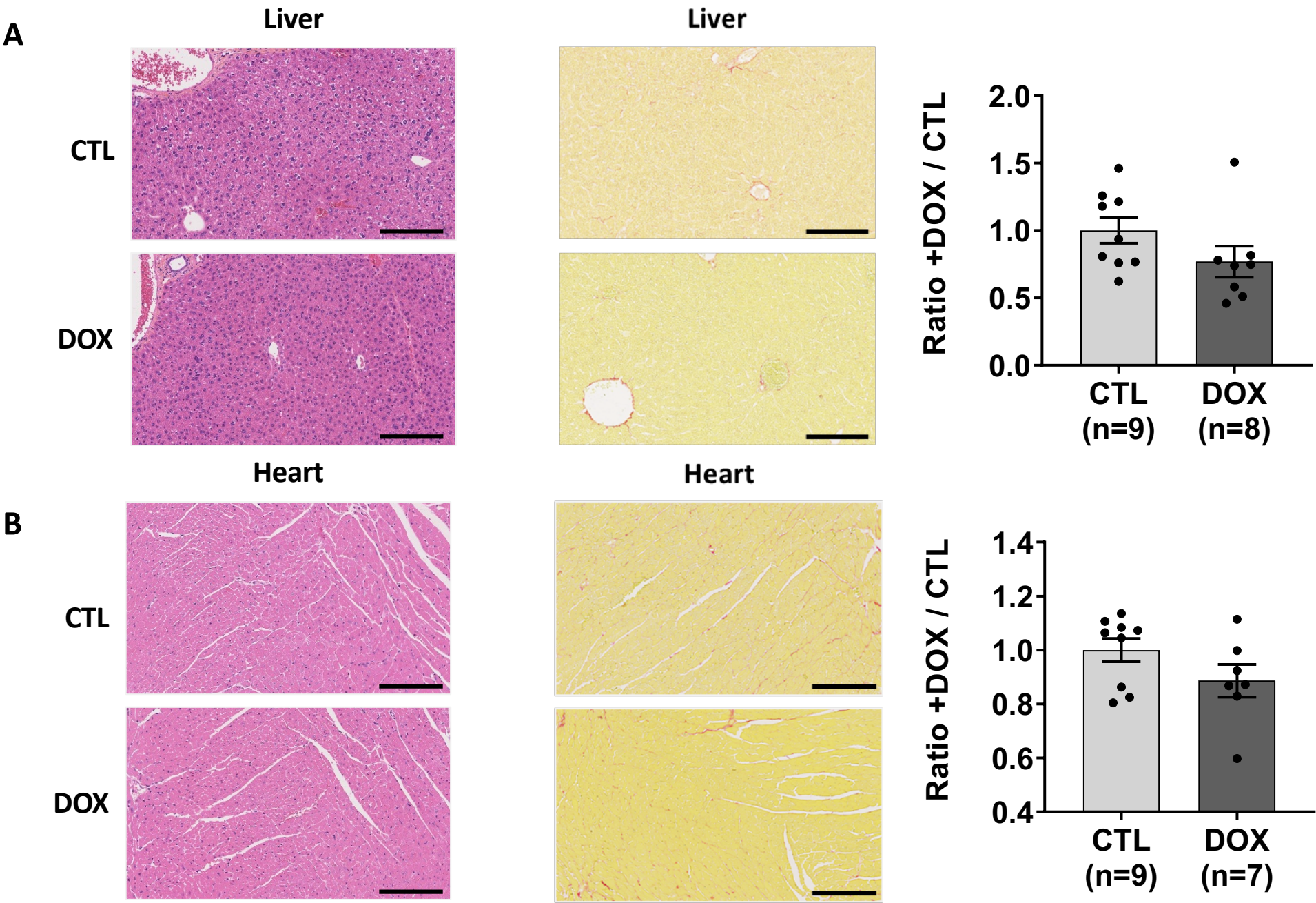

Supplement: Supplementary file 5 — Figure S3 [file ACEL-21-e13714-s003.pdf]

Supplementary Figure 4

A

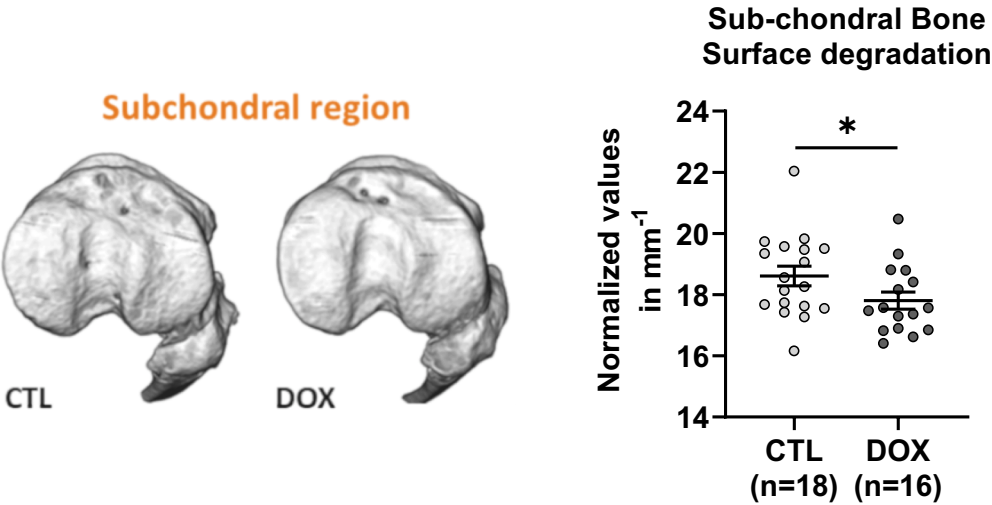

B

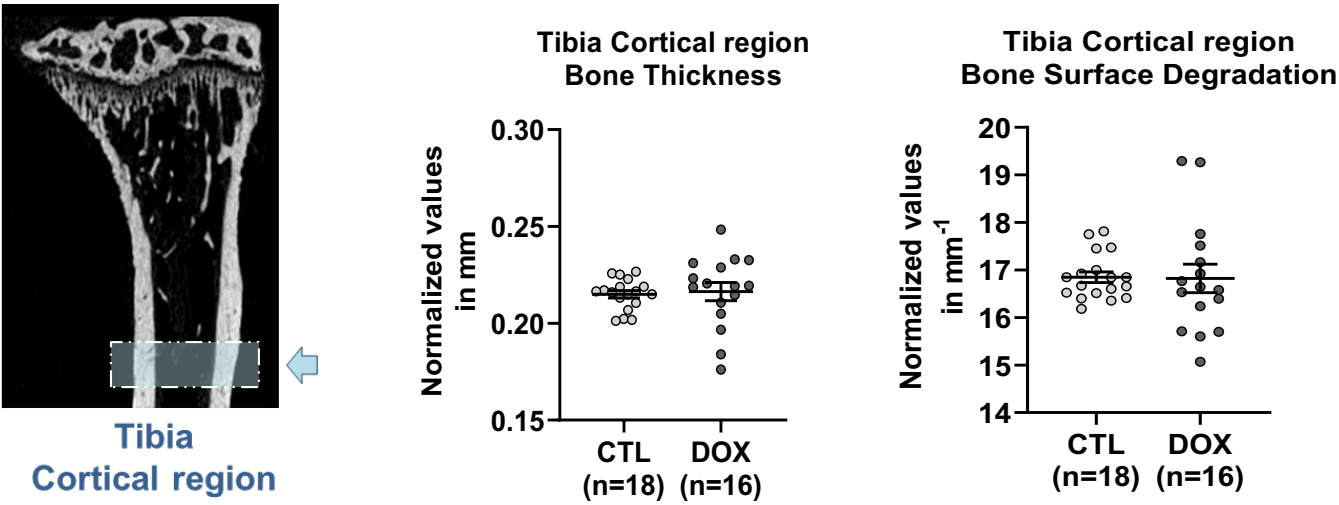

Supplement: Supplementary file 6 — Figure S4 [file ACEL-21-e13714-s015.pdf]

Supplementary Figure 5

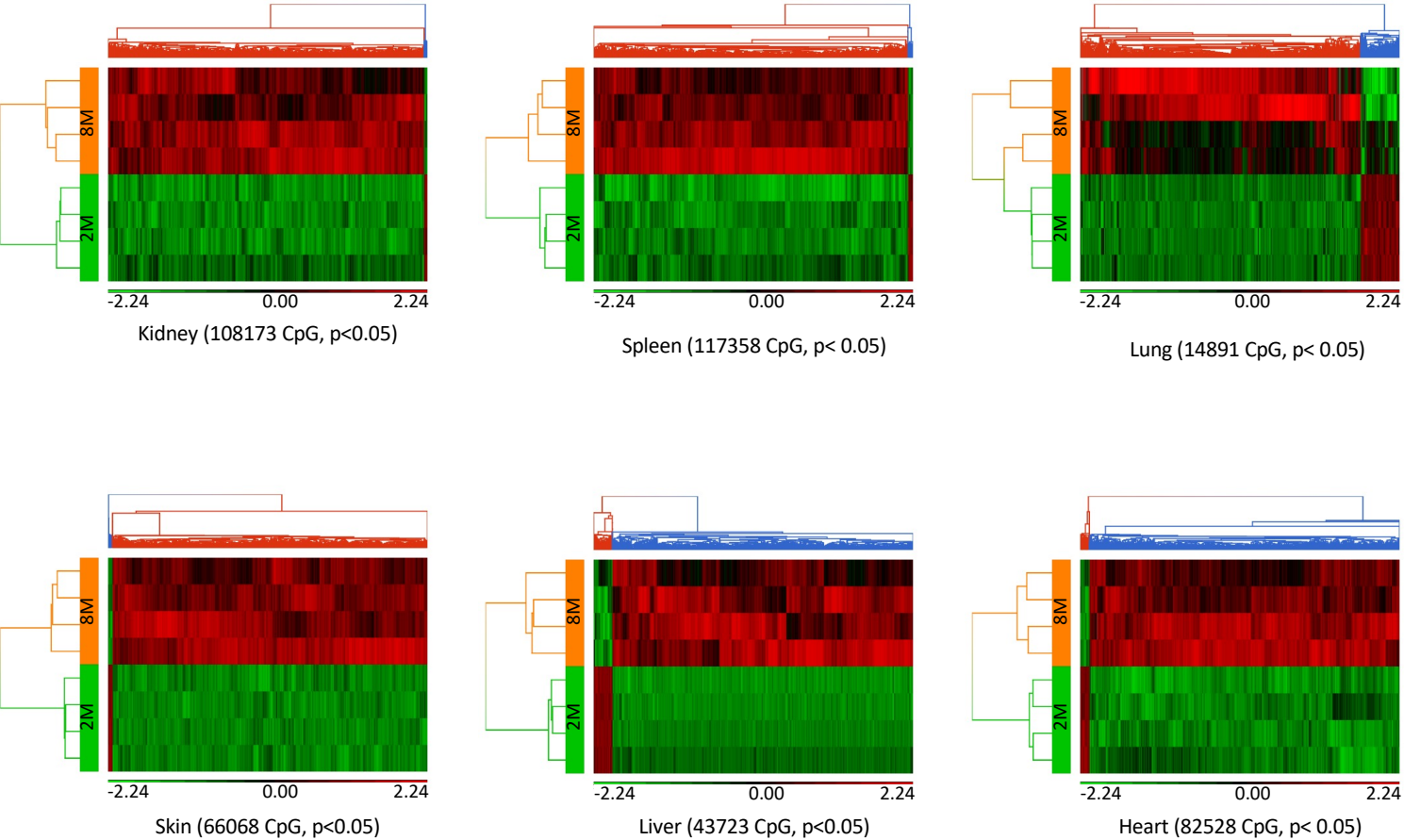

Supplement: Supplementary file 7 — Figure S5 [file ACEL-21-e13714-s004.pdf]

## Supplementary Figure 6

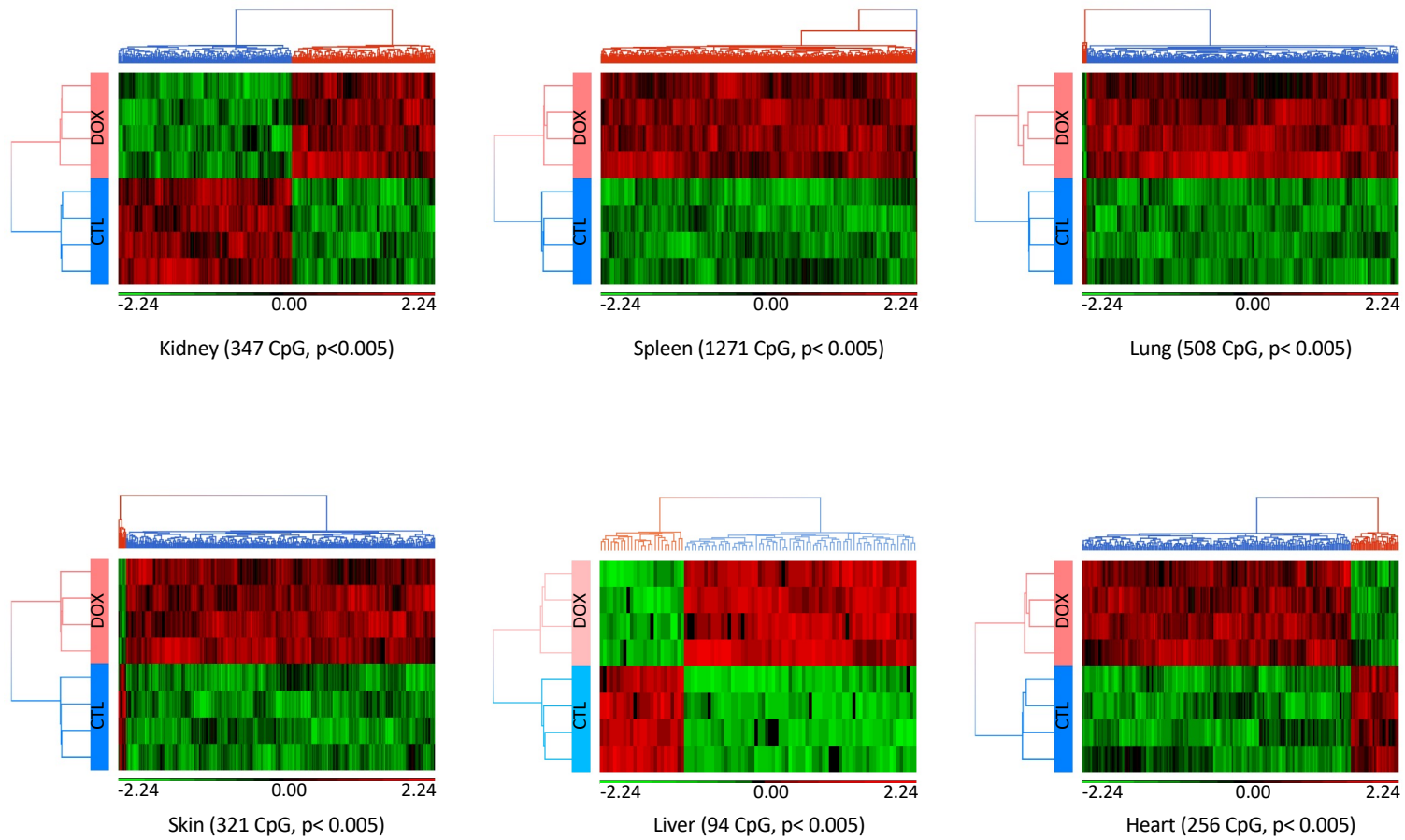

Supplement: Supplementary file 8 — Figure S6 [file ACEL-21-e13714-s006.pdf]

Supplementary Figure 7

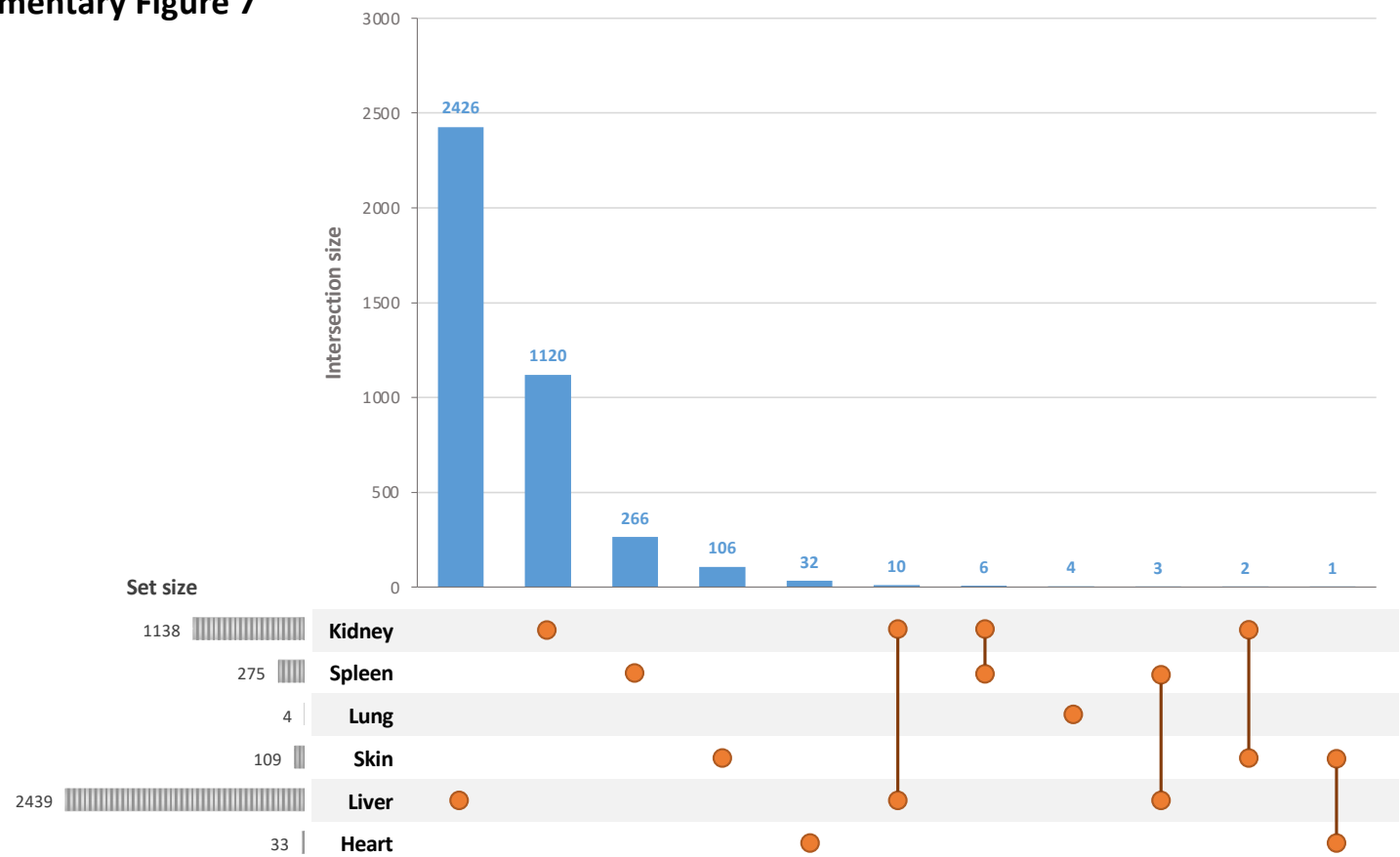

Supplement: Supplementary file 9 — Figure S7 [file ACEL-21-e13714-s014.pdf]

Supplementary Figure 8

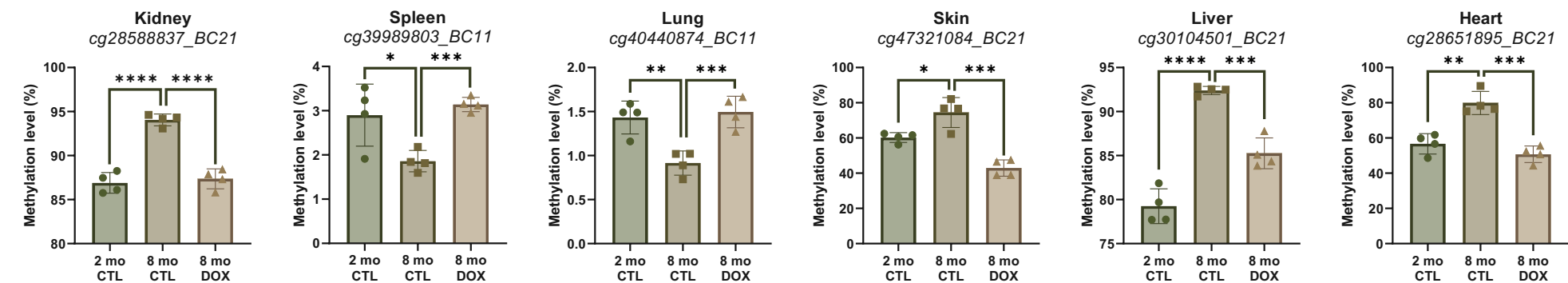

Supplement: Supplementary file 10 — Figure S8 [file ACEL-21-e13714-s001.pdf]

Supplementary Figure 9

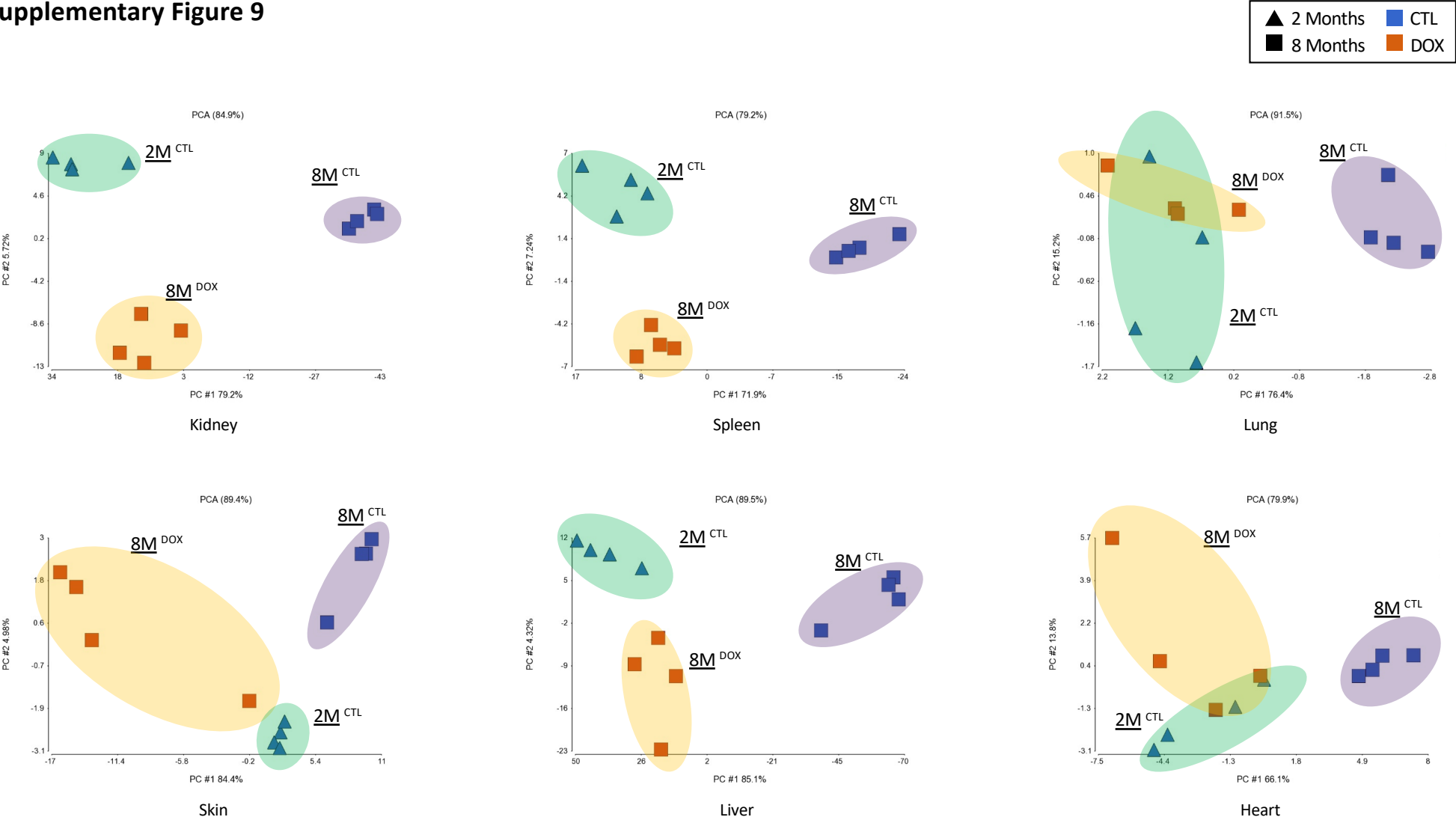

Supplement: Supplementary file 11 — Figure S9 [file ACEL-21-e13714-s009.pdf]

Supplementary Figure 10

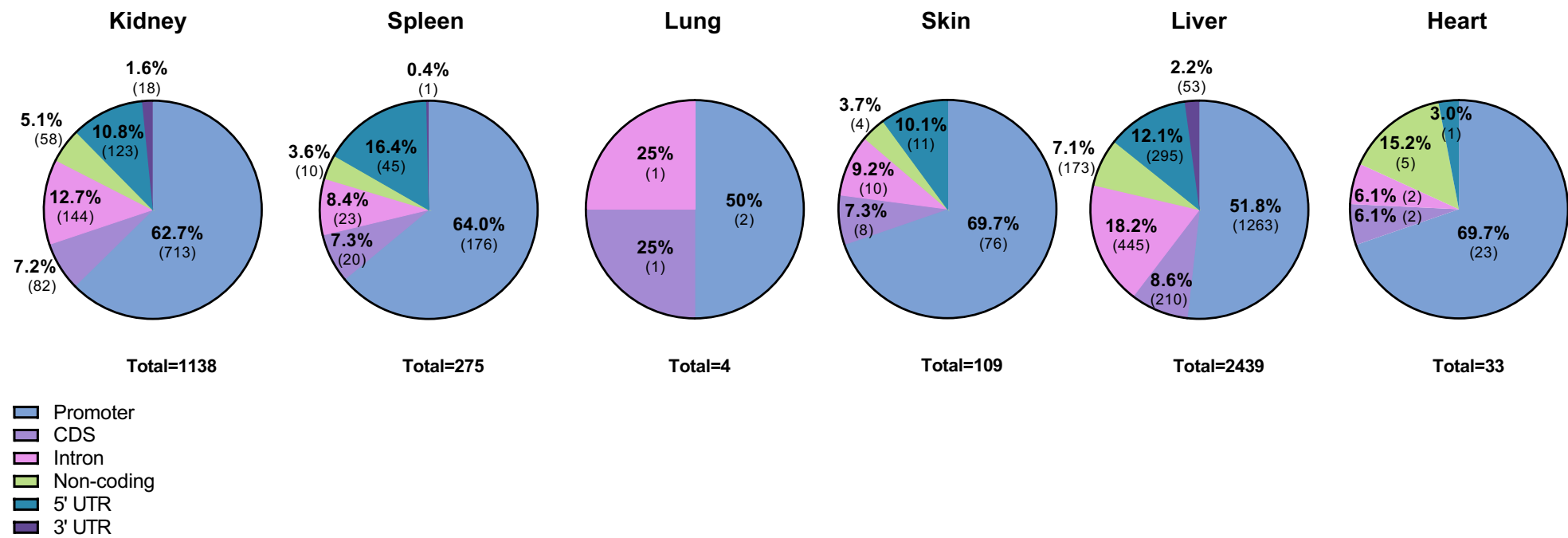

Supplement: Supplementary file 12 — Figure S10 [file ACEL-21-e13714-s011.pdf]

Supplementary Figure 11

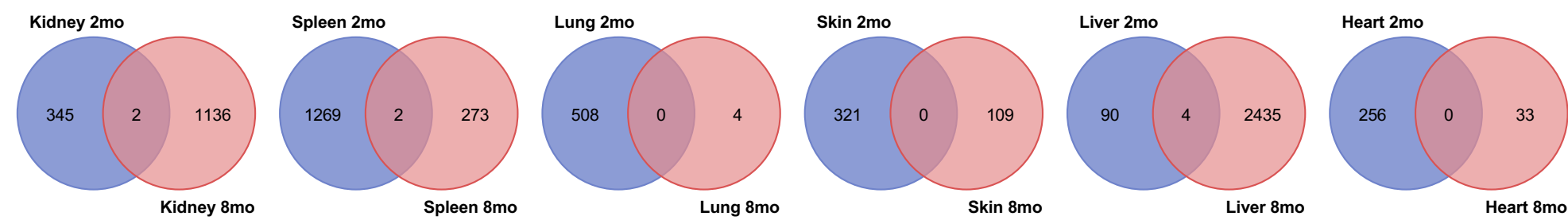

Supplement: Supplementary file 13 — Figure S11 [file ACEL-21-e13714-s016.pdf]

## Supplementary Figure 12

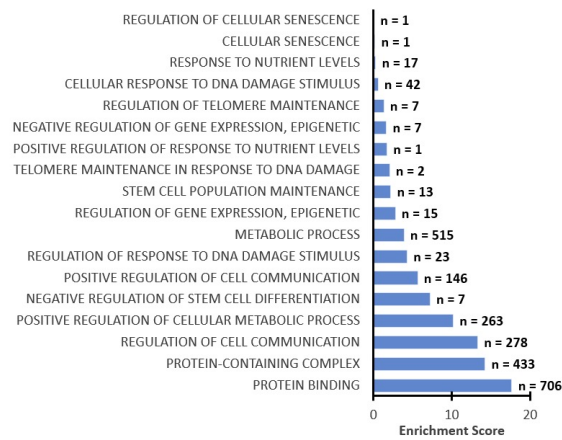

Kidney (1960 CpG, p<0.05)

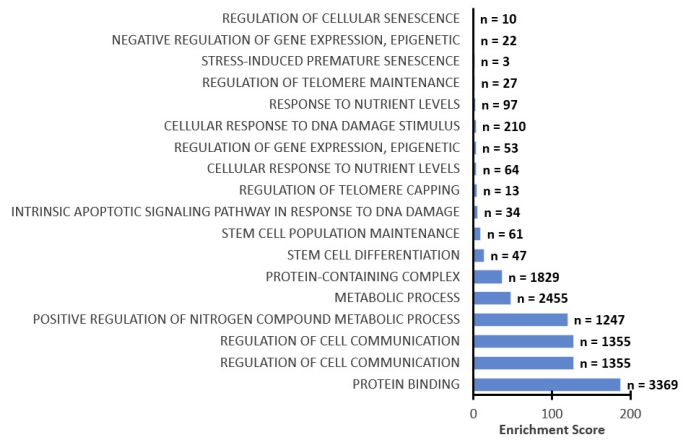

Spleen (11400 CpG, p<0.05)

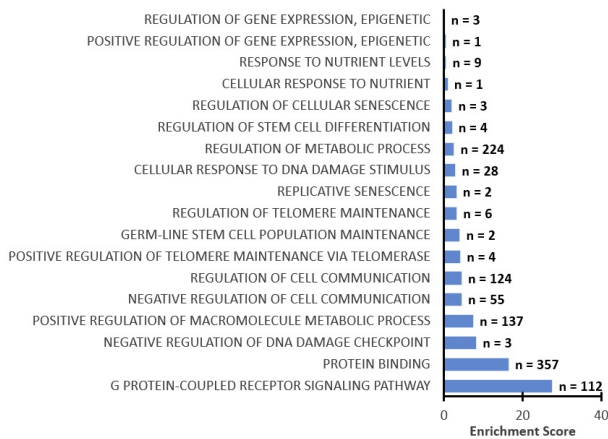

Lung (991 CpG, p<0.05)

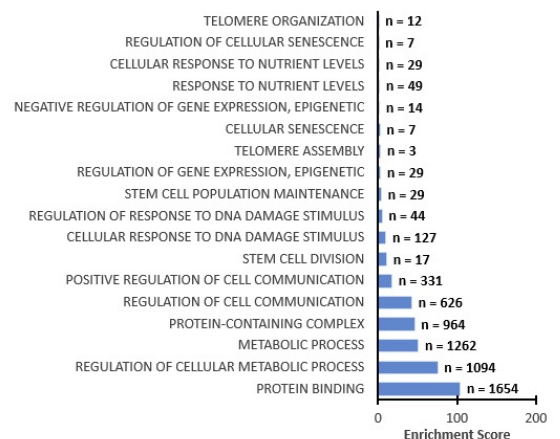

Skin (3496 CpG, p<0.05)

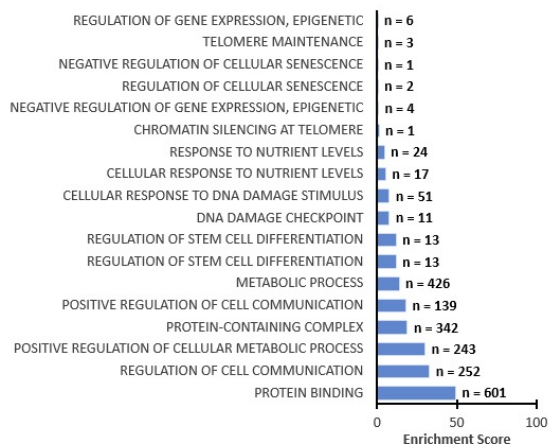

Liver (1266 CpG, p<0.05)

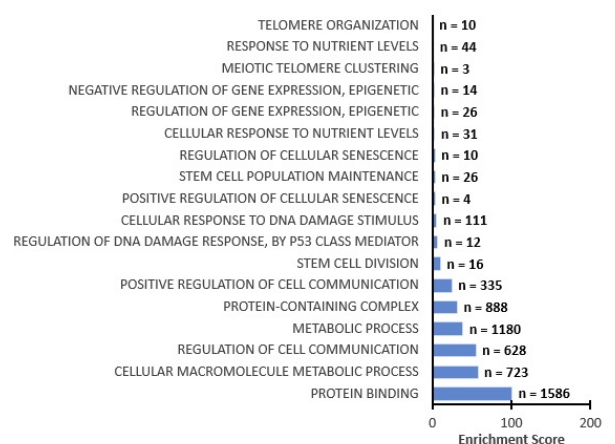

Heart (3863 CpG, p<0.05)

Supplement: Supplementary file 14 — Figure S12 [file ACEL-21-e13714-s007.pdf]

## Supplementary Figure 13

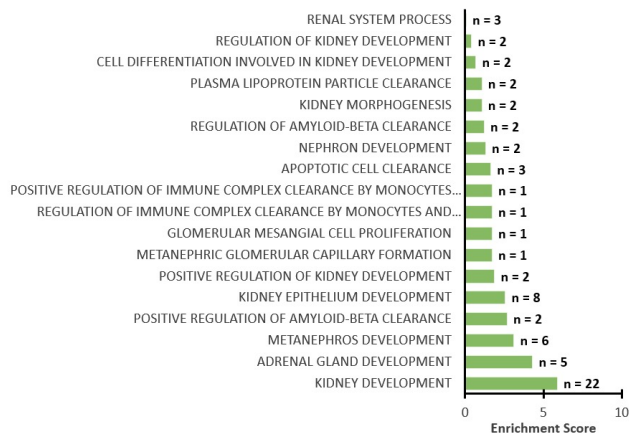

Kidney (1960 CpG,  $p < 0.05$ )

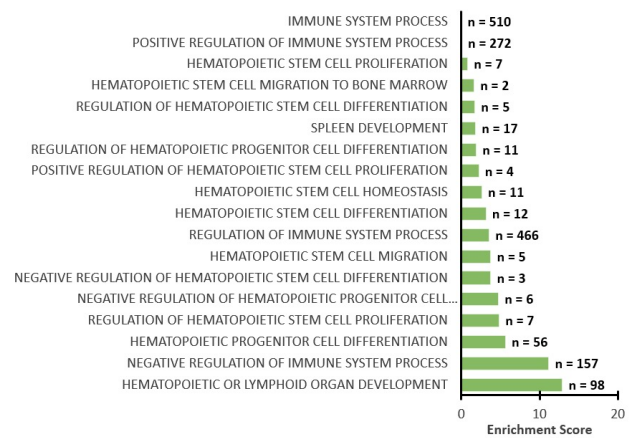

Spleen (11400 CpG,  $p < 0.05$ )

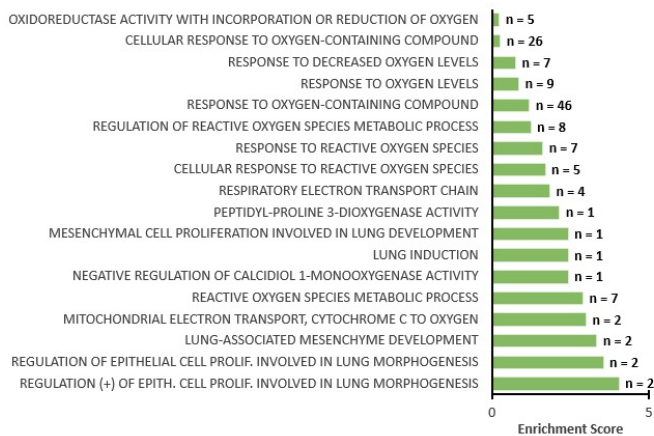

Lung (991 CpG,  $p < 0.05$ )

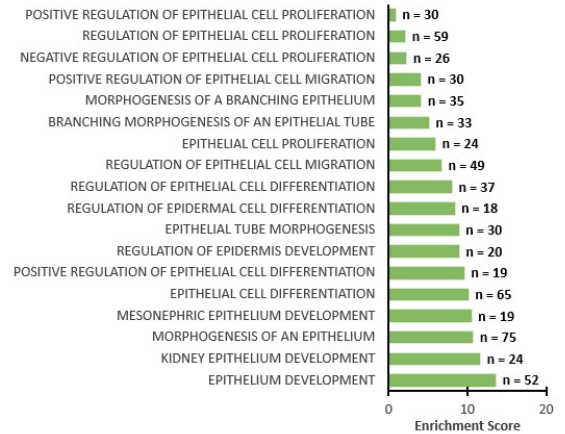

Skin (3496 CpG,  $p < 0.05$ )

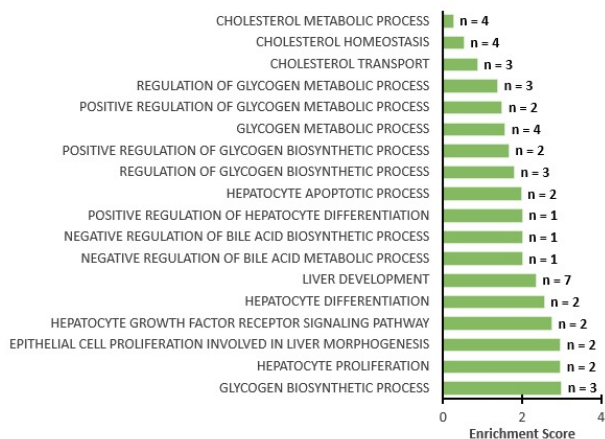

Liver (1266 CpG,  $p < 0.05$ )

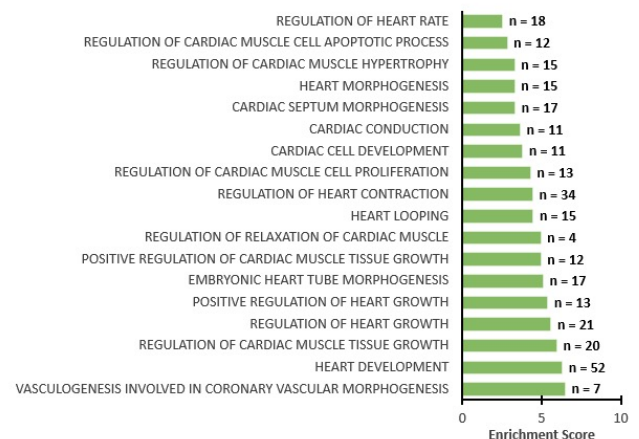

Heart (3863 CpG,  $p < 0.05$ )

Supplement: Supplementary file 15 — Figure S13 [file ACEL-21-e13714-s013.pdf]

## Supplementary Figure 14

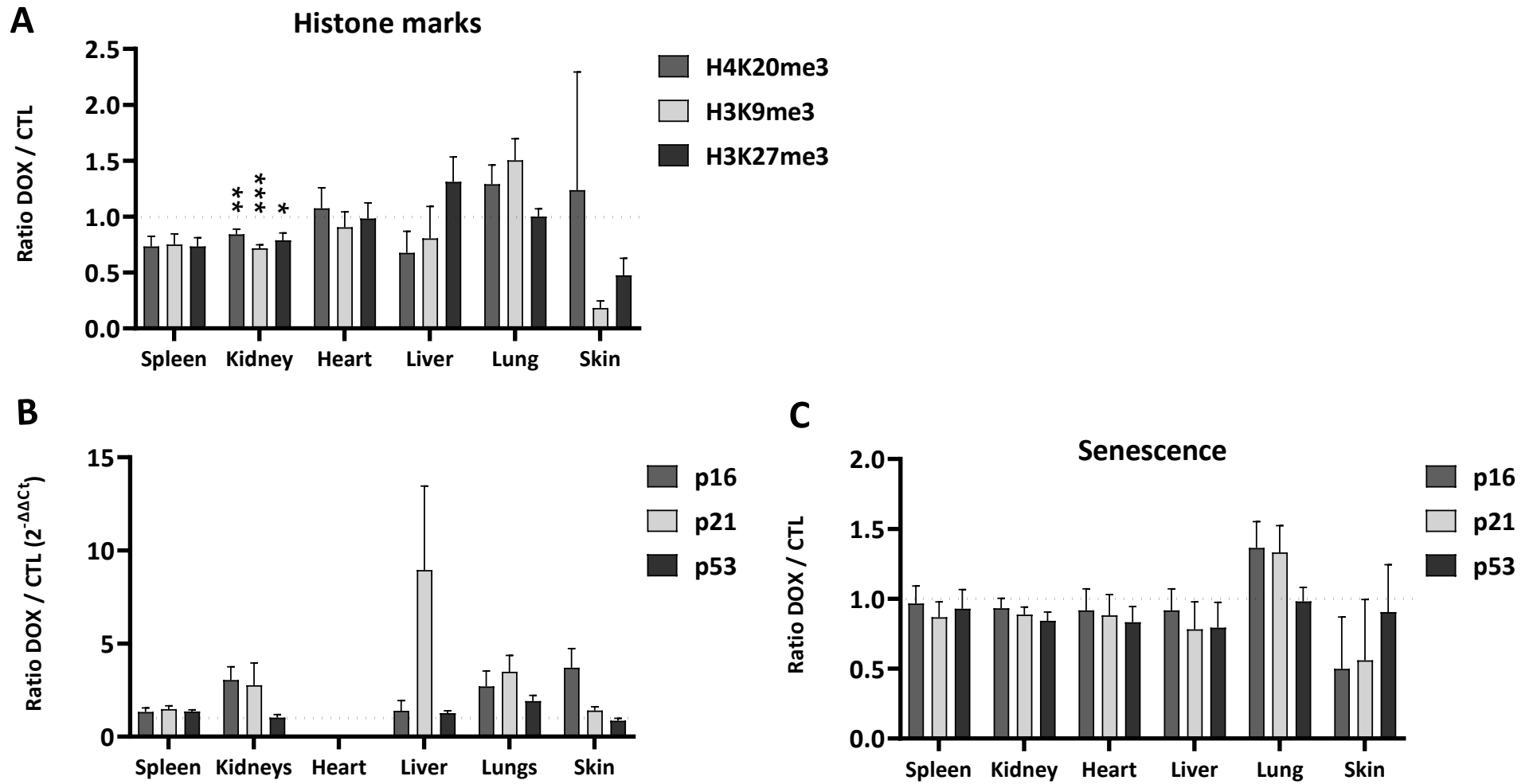

Supplement: Supplementary file 16 — Figure S14 [file ACEL-21-e13714-s008.pdf]
